# Heat-hypersensitive mutants of ryanodine receptor type 1 revealed by microscopic heating

**DOI:** 10.1073/pnas.2201286119

**Published:** 2022-08-04

**Authors:** Kotaro Oyama, Vadim Zeeb, Toshiko Yamazawa, Nagomi Kurebayashi, Fuyu Kobirumaki-Shimozawa, Takashi Murayama, Hideto Oyamada, Satoru Noguchi, Takayoshi Inoue, Yukiko U. Inoue, Ichizo Nishino, Yoshie Harada, Norio Fukuda, Shin’ichi Ishiwata, Madoka Suzuki

**Affiliations:** ^a^Quantum Beam Science Research Directorate, National Institutes for Quantum Science and Technology, Takasaki-shi, Gunma 370-1292, Japan;; ^b^PRESTO, Japan Science and Technology Agency, Kawaguchi-shi, Saitama 332-0012, Japan;; ^c^Department of Cell Physiology, The Jikei University School of Medicine, Minato-ku, Tokyo 105-8461, Japan;; ^d^Department of Physics, Faculty of Science and Engineering, Waseda University, Shinjuku-ku, Tokyo 169-8555, Japan;; ^e^Institute of Theoretical and Experimental Biophysics, Russian Academy of Sciences, Pushchino, Moscow Region 142290, Russia;; ^f^Core Research Facilities, The Jikei University School of Medicine, Minato-ku, Tokyo 105-8461, Japan;; ^g^Department of Molecular Physiology, The Jikei University School of Medicine, Minato-ku, Tokyo 105-8461, Japan;; ^h^Department of Pharmacology, Graduate School of Medicine, The University of Tokyo, Tokyo 113-0033, Japan;; ^i^Department of Cellular and Molecular Pharmacology, Juntendo University Graduate School of Medicine, Bunkyo-ku, Tokyo 113-8421, Japan;; ^j^Pharmacological Research Center, Showa University, Shinagawa-ku, Tokyo 142-8555, Japan;; ^k^Department of Neuromuscular Research, National Institute of Neuroscience, National Center of Neurology and Psychiatry, Kodaira-shi, Tokyo 187-8551, Japan;; ^l^Department of Biochemistry and Cellular Biology, National Institute of Neuroscience, National Center of Neurology and Psychiatry, Kodaira-shi, Tokyo 187-8502, Japan;; ^m^Institute for Protein Research, Osaka University, Suita, Osaka 565-0871, Japan

**Keywords:** heat-sensing, malignant hyperthermia, microheating, calcium channel, skeletal muscle

## Abstract

Thermoregulation is an important aspect of human homeostasis, and high temperatures pose serious stresses for the body. Malignant hyperthermia (MH) is a life-threatening disorder in which body temperature can rise to a lethal level. Here we employ an optically controlled local heat-pulse method to manipulate the temperature in cells with a precision of less than 1 °C and find that the mutants of ryanodine receptor type 1 (RyR1), a key Ca^2+^ release channel underlying MH, are heat hypersensitive compared with the wild type (WT). We show that the local heat pulses induce an intracellular Ca^2+^ burst in human embryonic kidney 293 cells overexpressing WT RyR1 and some RyR1 mutants related to MH. Fluorescence Ca^2+^ imaging using the endoplasmic reticulum–targeted fluorescent probes demonstrates that the Ca^2+^ burst originates from heat-induced Ca^2+^ release (HICR) through RyR1-mutant channels because of the channels’ heat hypersensitivity. Furthermore, the variation in the heat hypersensitivity of four RyR1 mutants highlights the complexity of MH. HICR likewise occurs in skeletal muscles of MH model mice. We propose that HICR contributes an additional positive feedback to accelerate thermogenesis in patients with MH.

## Significance

Malignant hyperthermia (MH) is a life-threatening disorder caused largely by mutations in ryanodine receptor type 1 (RyR1) Ca^2+^-release channels. Enhanced Ca^2+^ release through the mutant channels induces excessive heat development upon exposure to volatile anesthetics. However, the mechanism by which Ca^2+^ release is accelerated at an elevated temperature is yet to be identified. Fluorescence Ca^2+^ imaging with rapid heating by an infrared laser beam provides direct evidence that heat induces Ca^2+^ release through the RyR1 channel. And the mutant channels are more heat sensitive than the wild-type channels, thereby causing an increase in the cytosolic Ca^2+^ concentration in mutant cells. It is likely that the heat-induced Ca^2+^ release participates as an enhancer in the cellular mechanism of MH.

Malignant hyperthermia (MH) is a life-threatening disorder, triggered by volatile anesthetics or depolarizing muscle relaxants (1, 2). In MH, heat markedly affects the processes governing cellular thermogenesis, and the body temperature rises well beyond normal (sometimes >42 °C). Typical symptoms of MH are associated with elevated body temperatures above 39 °C; these symptoms can be fatal unless treated immediately.

MH is caused by mutations in ryanodine receptor type 1 (RyR1) Ca^2+^-release channels, dihydropyridine receptors (sarcolemmal slow, voltage-gated Ca^2+^ channels), and Src-homologous-3 and cysteine-rich, domain-containing protein 3 in skeletal muscles (1, 3–5). Most human MH-associated mutations have been identified in the RyR1 gene. Under physiological conditions, Ca^2+^ is released from the sarcoplasmic reticulum (SR) through RyR1 and causes reversible sarcomere contraction during excitation–contraction coupling. In skeletal muscles expressing these mutants, however, anesthesia-enhanced Ca^2+^ release from the SR elevates the intracellular Ca^2+^ concentration ([Ca^2+^]_i_) and causes uncontrolled hypermetabolism and hyperthermia (2). It has been reported that environmental heat stress likewise triggers MH-like phenomena in knock-in mice expressing RyR1 mutants (6–8) and increases [Ca^2+^]_i_ in single skeletal muscle fibers expressing the mutants (9). These studies suggest mutual amplification between Ca^2+^ release and thermogenesis (i.e., a positive feedback loop) in the progression of MH. However, the intrinsic feature of this positive feedback loop remains elusive because it is still unclear how elevated body temperature affects Ca^2+^ release.

In the present study, by applying optically controlled local heat pulses (10–14) to human embryonic kidney (HEK) 293 cells overexpressing RyR1 mutants related to MH, we investigated the heat sensitivities of the mutants via fluorescence imaging of Ca^2+^ in the intracellular space as well as in the endoplasmic reticulum (ER). Our quantitative analysis demonstrated that the heat-induced Ca^2+^ release (HICR) mechanism contributes an additional positive feedback loop between Ca^2+^ and thermogenesis. The RyR1 mutants related to MH displayed greater heat sensitivity than did wild-type (WT) RyR1, and the sensitivity varied among mutants. Likewise, skeletal muscles from MH model mice were found to be more heat sensitive than those from WT mice. These findings led us to propose the following cascade regarding the progression of MH: 1) MH is triggered by Ca^2+^ release from the SR via the RyR1 mutants upon exposure to volatile anesthetics; 2) a small magnitude of heat stress causes Ca^2+^ release via the heat-hypersensitive RyR1 mutants; 3) the released Ca^2+^ causes hypermetabolism and hyperthermia, accelerating Ca^2+^ release; and then 4) the thermogenic cascade possibly results in lethal hyperthermia if not treated properly.

## Results

### Heat-Induced Ca^2+^ Bursts in MH Mutants.

**Fig. 1.**
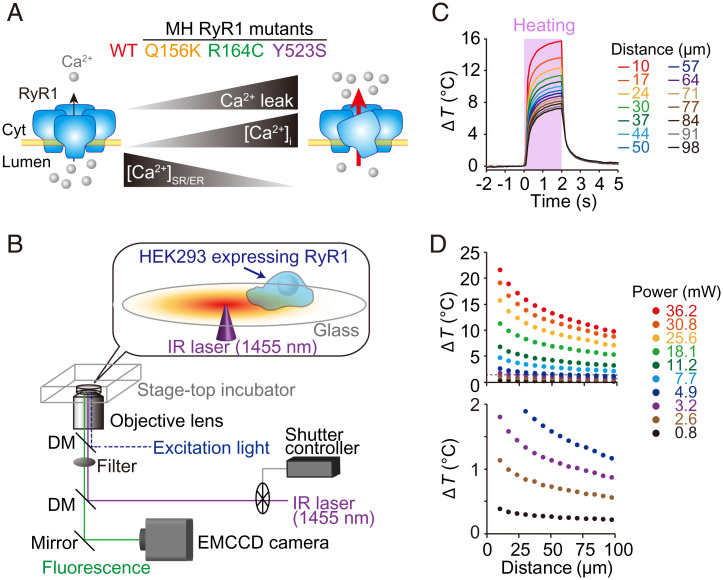
Experimental design to investigate heat sensitivities of various MH mutants of RyR1. (*A*) Properties of MH mutants of RyR1 investigated in the present study. Ca^2+^ leakage through RyR1 mutants (higher rank) is greater than that through WT receptors (lowest rank). [Ca^2+^]_i_ of cells expressing RyR1 mutants is higher than that of HEK 293 cells expressing WT RyR1. The [Ca^2+^] in sarco/endoplasmic reticulum ([Ca^2+^]_SR/ER_) is depleted because of Ca^2+^ leakage. The rank order of activity was based on the literature (15, 16). (*B*) Schematic illustration of the fluorescence microscopy setup used in the present study. A 1,455-nm IR laser beam was guided to the sample stage by a dichroic mirror (DM) and an objective lens, and focused on the medium. The temperature in the field of view was elevated locally (*Materials and Methods*). Sample temperature was controlled by a stage-top incubator. (*C*) Time courses of Δ*T* at various distances from the heat source. Δ*T* was measured on the surface of a glass base dish by thermal quenching of the temperature-sensitive dye europium (III) thenoyltrifluoroacetonate trihydrate. The pink vertical bar indicates the period of heating. Laser power was 25.6 mW. (*D*) Temperature gradients formed by various laser powers. Bottom panel shows the enlarged view of Δ*T*s between 0 and 2 °C. EMCCD, electron-multiplying charge-coupled device.

First, we quantified the heat pulse–induced Ca^2+^ release in HEK293 cells expressing either WT or RyR1 mutants. We selected three mutants across the rank order of the activity of RyR1 mutants in the N-terminal region, with Q156K, R164C, and Y523S having the lowest, intermediate, and highest ranking, respectively (i.e., WT < Q156K < R164C < Y523S) (15–17) (Fig. 1*A*). The rank order of activity was determined previously based on the magnitude of Ca^2+^ leakage (16) (i.e., a higher rank indicates higher cytosolic [Ca^2+^] and hence lower [Ca^2+^] in the SR or ER) (15, 16, 18, 19).

Heat stimulation was applied using a focused 1,455-nm near-infrared (IR) laser beam (10–14, 20), and fluorescence Ca^2+^ imaging was performed simultaneously (Fig. 1*B*). Changes in local temperature were measured by a luminescent thermometer nanosheet placed on a glass base dish without cells (11–13). The temperature in the field of view increased immediately (<100 ms) upon heat stimulation (for 2 s), and it returned to the original level (recooling) when the stimulation ceased after heating (Fig. 1*C*). The amplitude of the change in temperature (Δ*T*) was adjustable down to 1 °C or lower by reducing the laser power or by increasing the distance from the point at which laser irradiation was focused (Fig. 1*D* and *SI Appendix*, Fig. S1). The present experimental system, therefore, allowed us to apply the heat pulse to cells with various Δ*T*s in the same field of view (138 µm × 138 µm).

**Fig. 2.**
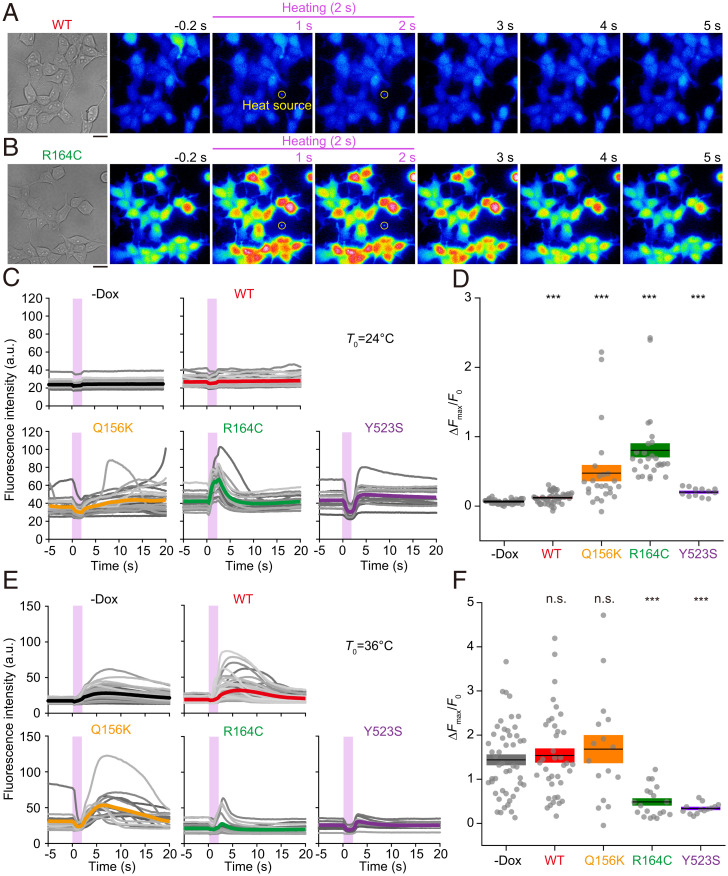
Heat-induced Ca^2+^ bursts in HEK293 cells expressing RyR1 mutants. (*A* and *B*) Bright-field and fluorescence images of fluo-4-loaded HEK293 cells expressing WT RyR1 (*A*) and R164C (*B*). Background intensity was slightly increased during heating due to IR laser-beam scattering (Movie S1). Yellow circles indicate the position of the heat source. Scale bars, 20 µm. (*C*) Changes in the fluorescence intensity of fluo-4 in cells without induction of RyR1 expression(−Dox) as a control, or with induction of the expression of WT RyR1, or the mutants (Q156K, R164C, or Y523S). Each gray line represents an individual cell. Changes in the background intensities caused by IR laser-beam scattering were subtracted from the fluo-4 signals (for raw data, see *SI Appendix*, Fig. S2*A*). Thick, colored lines indicate the average intensities. Pink vertical bars indicate the period of heating. In contrast to R164C cells, Q156K and Y523S cells showed a decrease in the fluorescence intensity during heating. (*D*) Δ*F*_max_/*F*_0_ of fluo-4 during the 20 s after the onset of heating. Horizontal bars and boxes indicate means ± SEM. Statistical significance was determined by comparison with −Dox cells (*n* = 40) using the Steel test. ***P < 0.001. WT, *n* = 43 and *P* = 3.5 × 10^−4^; Q156K, *n* = 25 and *P* = 5.3 × 10^−8^; R164C, *n* = 27 and *P* = 1.5 × 10^−11^; Y523S, *n* = 12 and *P* = 1.9 × 10^−6^. Laser power, 25.6 mW; Δ*T* = 10 ± 2 °C; *T*_0_ = 24 °C. (*E*) Time course of changes in the fluorescence intensity of fluo-4 in HEK293 cells at 36 °C. Changes in the background intensities caused by IR laser beam scattering were subtracted from the fluo-4 signals (for raw data, see *SI Appendix*, Fig. S2*B*). (*F*) Δ*F*_max_/*F*_0_, analyzed from data in (*E*). Statistical significance was determined by comparison with −Dox cells (*n* = 49) using the Steel test. ***P < 0.001. n.s., not significant. WT, *n* = 38 and *P* = 0.99; Q156K, *n* = 16 and *P* = 0.97; R164C, *n* = 19 and *P* = 1.9 × 10^−5^; Y523S, *n* = 13 and *P* = 1.9 × 10^−5^. Laser power, 25.6 mW; Δ*T* = 10 ± 1 °C; *T*_0_ = 36 °C. a.u., arbitrary units.

No [Ca^2+^]_i_ changes were observed in control cells with no induced RyR1 expression (−doxycycline [−Dox]; i.e., cells expressing endogenous Ca^2+^ channels) or in cells expressing WT RyR1 when heat pulses of Δ*T =* 10 ± 2 °C were applied at the base temperature *T*_0_ = 24 °C (Fig. 2*A* and Movie S1). In contrast, there were rapid (<∼500 ms) and large [Ca^2+^]_i_ increases (i.e., Ca^2+^ bursts) upon heating, and the Ca^2+^ bursts were sustained until recooling in most R164C cells in the field of view (Fig. 2*B* and Movie S2). In Q156K and Y523S cells, [Ca^2+^]_i_ decreased during heating, and Ca^2+^ bursts were observed after the onset of recooling (Fig. 2*C* and *SI Appendix*, Fig. S2*A* and Movies S3 and S4). The decrease in [Ca^2+^]_i_ during heating in those cells (apparent, likewise, in WT cells in Fig. 2*A* and Movie S1) is likely attributable to heat-activated Ca^2+^ uptake via activation of sarco/endoplasmic reticulum Ca^2+^-ATPase (SERCA), as suggested by us (11, 21) and others (22). Western blotting analyses revealed that the expression levels of SERCA were similar across the cell lines (*SI Appendix*, Fig. S3). Therefore, the differential magnitudes of the heat-induced decrease in [Ca^2+^]_i_ across the mutant lines are not likely coupled with a difference in the SERCA expression levels. The maximum changes in the Ca^2+^ bursts of fluorescence intensity (Δ*F*_max_)/*F*_0_ during the 20 s after the onset of heating were significantly larger in WT, Q156K, R164C, and Y523S cells than in −Dox cells (Fig. 2*D*). The experiments were likewise performed at the physiological *T*_0_ = 36 °C with the same Δ*T = *10 ± 1 °C. The Ca^2+^ bursts observed in Q156K, R164C, and Y523S cells were similar to those at *T*_0_ = 24 °C, whereas those in Q156K and R164C were initiated during heating and sustained until recooling (Fig. 2*E* and *SI Appendix*, Fig. S2*B*). Significant Ca^2+^ bursts were observed in −Dox and WT cells with mean Δ*F*_max_/*F*_0_ values comparable to that of Q156K (Fig. 2*F*). To summarize, Δ*F*_max_/*F*_0_ and the fraction of cells exhibiting Ca^2+^ bursts were dependent on the RyR1 mutants, as well as on *T*_0_.

### Heat-Induced Ca^2+^ Release through RyR1s.

**Fig. 3.**
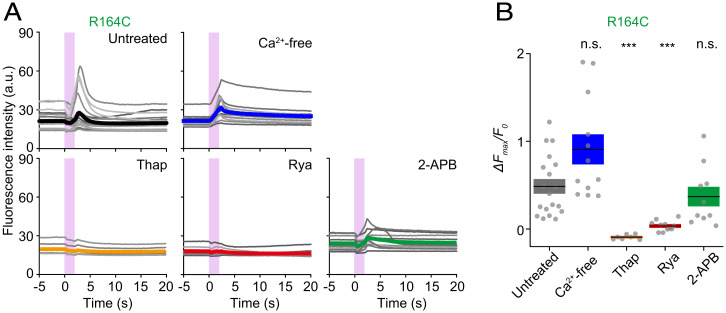
Heat-induced Ca^2+^ bursts in R164C cells under various conditions. (*A*) Time courses of the fluorescence intensity of fluo-4 in HEK293 cells expressing R164C in an untreated condition, in Ca^2+^-free solution (Ca^2+^-free), and in the presence of 2 µM thapsigargin (Thap), 100 µM ryanodine (Rya), or 100 µM 2-APB. Each gray line represents an individual cell. Changes in the background intensities caused by IR laser-beam scattering were subtracted from the fluo-4 signals (for raw data, see *SI Appendix*, Fig. S4*A*). Thick, colored lines represent averages. Pink vertical bars indicate the period of heating. (*B*) Δ*F*_max_/*F*_0_. Horizontal bars and boxes indicate means ± SEM. Statistical significance was examined by comparison with the untreated cells (*n* = 19) using the Steel test. ***P < 0.001. n.s., not significant. Ca^2+^-free: *n* = 12, *P* = 0.11; Thap, 4.7 × 10^−4^ (*n* = 7); Rya, 4.1 × 10^−5^ (*n* = 11); and 2-APB, 0.83 (*n* = 10). Laser power, 25.6 mW; Δ*T* = 10 ± 1 °C; *T*_0_ = 36 °C. Note that despite the lack of a significant difference in maximal amplitudes, the kinetics of the fluo-4 intensity in Ca^2+^-free solution differed from that in untreated-cells. a.u., arbitrary units.

The RyR1 mutant−dependent variation in heat-induced Ca^2+^ bursts indicates that the ER functions as a major Ca^2+^ source, and Ca^2+^ flows through RyR1 channels from the ER lumen to the cytosol. Therefore, we undertook this study to determine the primary Ca^2+^ source in cells expressing WT RyR1 or R164C, as well as −Dox cells (Fig. 3*A* and *SI Appendix*, Fig. S4 *A* and *B*) at *T*_0_ = 36 °C. R164C was chosen as the RyR1 mutant of the middle-rank order. To compare the mutant response with its WT, WT RyR1 cells were examined. −Dox was used to examine the contribution of endogenous Ca^2+^ channels. The Ca^2+^ burst was preserved in all WT, R164C, and −Dox cells in Ca^2+^-free medium, whereas the burst was suppressed when Ca^2+^ was depleted from the ER by 2 µM thapsigargin (an inhibitor of SERCA). These results indicate that the Ca^2+^ source for the heat-induced Ca^2+^ burst is the ER, not the extracellular space.

The major Ca^2+^ channels that function physiologically on the ER membrane are ryanodine receptors (RyRs) and inositol trisphosphate receptors (IP_3_Rs). Here, Ca^2+^ bursts in WT RyR1 and R164C cells were blocked by 100 µM ryanodine (an inhibitor of RyRs) (Fig. 3 *A* and *B* and *SI Appendix*, Fig. S4 *B* and *C*), indicating that these RyR1 channels operate in Ca^2+^ bursts. The suppression of Ca^2+^ bursts by ryanodine was much less effective in −Dox cells than in WT RyR1 and R164C cells (*SI Appendix*, Fig. S4 *B* and *D*), providing confirmatory evidence that the contribution of the endogenous RyRs (23) is minor at best. The contribution of endogenous IP_3_Rs was likewise examined because we previously reported that IP_3_Rs play a major role in heat-induced Ca^2+^ bursts upon recooling in HeLa (21) and WI-38 (11) cells. Accordingly, we found a significant suppression of Ca^2+^ bursts in −Dox cells by 100 µM 2-aminoethoxydiphenyl borate [2-APB; an unspecific inhibitor of IP_3_Rs (24–26), of which the half maximal inhibition concentration to IP_3_Rs is 42 µM (27)] (*SI Appendix*, Fig. S4 *B* and *D*), demonstrating a major contribution of IP_3_Rs to Ca^2+^ bursts in −Dox cells. The relatively large Ca^2+^ bursts in −Dox cells at *T*_0_ = 36 °C compared with those at *T*_0_ = 24 °C (Fig. 2 *C* and *E*) likely reflect the *T*_0_-dependent heat sensitivity of IP_3_R, as previously demonstrated by us in other cell lines (11, 21). Although the peak intensity of fluo-4 (Δ*F*_max_/*F*_0_) was reduced by 2-APB in WT RyR1 cells, Ca^2+^ bursts were observed (*SI Appendix*, Fig. S4 *B* and *C*). Furthermore, Ca^2+^ bursts were not significantly suppressed by 2-APB in R164C cells (Fig. 3 *A* and *B*). It has been reported that 2-APB likewise inhibits transient receptor potential canonical channels (28–30). Therefore, our results strongly suggest that the contribution of Ca^2+^ influx via transient receptor potential canonical channels (31, 32) is not essential in heat-induced Ca^2+^ bursts in R164C cells. We therefore conclude that overexpressed WT or mutant RyR1 channels play a dominant role in producing Ca^2+^ bursts.

### ER as a Source of Heat-Induced Ca^2+^ Bursts.

**Fig. 4.**
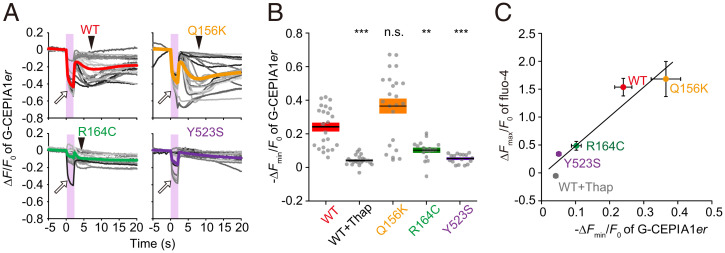
Heat-induced Ca^2+^ release from the ER. (*A*) Δ*F*/*F*_0_ of G-CEPIA1*er* in cells expressing WT RyR1, or the mutants (Q156K, R164C, or Y523S). Data in *SI Appendix*, Fig. S5*A* were analyzed and plotted. *n* = 23, 23, 17, and 17 cells for WT, Q156K, R164C, and Y523S, respectively. Each gray line represents an individual cell. Changes in the background intensities caused by IR laser-beam scattering were subtracted from the G-CEPIA1*er* signals (for raw data, see *SI Appendix*, Fig. S5*A*). Thick, colored lines represent averages. Pink vertical bars indicate the period of heating. The second decrease in Δ*F*/*F*_0_ is indicated by an arrowhead in the averaged data (thick curves for WT, Q156K, and R164C). The decrease in Δ*F*/*F*_0_ in some cells (indicated by an open arrow) during heating is presumably the result of thermal quenching of G-CEPIA1*er*. (*B*) Minimum relative change in G-CEPIA1*er* fluorescence intensity −Δ*F*_min_/*F*_0_ after heating for 2.4 to 10 s. Statistical significance was determined by comparison with WT using the Steel test. **P < 0.01; ***P < 0.001. n.s., not significant. WT + thapsigargin (Thap), *P* = 9.0 × 10^−8^ (*n* = 22); Q156K, *P* = 0.087; R164C, *P* = 0.0037 (R164C); and Y523S, *P* = 9.8 × 10^−7^. Horizontal bars and boxes indicate means ± SEM. (*C*) Relationship between the change in [Ca^2+^]_er_ (−Δ*F*_min_/*F*_0_ of G-CEPIA1*er*) and [Ca^2+^]_i_ (Δ*F*_max_/*F*_0_ of fluo-4; Fig. 2*F*). Correlation coefficient (*R*) was 0.96 (*P* = 0.011). Laser power, 25.6 mW; Δ*T* = 10 ± 1 °C; *T*_0_ = 36 °C.

To further strengthen the idea that the ER is the source of Ca^2+^ in the heat-induced Ca^2+^ burst, we analyzed the [Ca^2+^] in the lumen of ER ([Ca^2+^]_ER_) via expression of the ER-targeted fluorescent Ca^2+^ probe G-CEPIA1*er* (33) in WT RyR1, Q156K, R164C, and Y523S cells. In WT RyR1, the fluorescence intensity of G-CEPIA1*er* was decreased during the heat pulse of Δ*T* = 10 ± 1 °C, and then recovered immediately upon recooling (Fig. 4*A* and *SI Appendix*, Fig. S5*A* and Movie S5). The quick recovery suggests that the decrease during the heat pulse was due to thermal quenching of G-CEPIA1*er* fluorescence. After heating, the signal decreased and reached the minimum at ∼5 s after the cessation of the heat pulse (as indicated by the arrowheads in Fig. 4*A*), which recovered (i.e., increased) gradually. The initial value *F*_0_ of G-CEPIA1*er*, as well as the second decrease (the arrowheads in Fig. 4*A*), was significantly reduced when Ca^2+^ was depleted from the ER lumen by 2 µM thapsigargin (Fig. 4*B* and *SI Appendix*, Fig. S5 *A* and *B*). Furthermore, the second decrease appeared to coincide with the Ca^2+^ burst in the cytosol (Fig. 2*E*). Therefore, the second decrease of G-CEPIA1*er* fluorescence is likely to represent the decrease in [Ca^2+^]_ER_. Individual values for the [Ca^2+^]_ER_ decrease in Q156K cells were comparatively dispersed and, hence, the mean value was not significantly different from that in WT cells, whereas the decrease in R164C or Y523S cells was significantly lower than that in WT cells (Fig. 4*B*). These results demonstrate a positive correlation between the amplitude of [Ca^2+^]_ER_ decrease (−Δ*F*_min_/*F*_0_ of G-CEPIA1*er*) and that of the Ca^2+^ burst (Δ*F*_max_/*F*_0_ of fluo-4) (Fig. 4*C*), strengthening our conclusion that the heat-induced Ca^2+^ burst arises from Ca^2+^ release from the ER through RyR1.

### Variation in Heat Sensitivity between RyR1s.

**Fig. 5.**
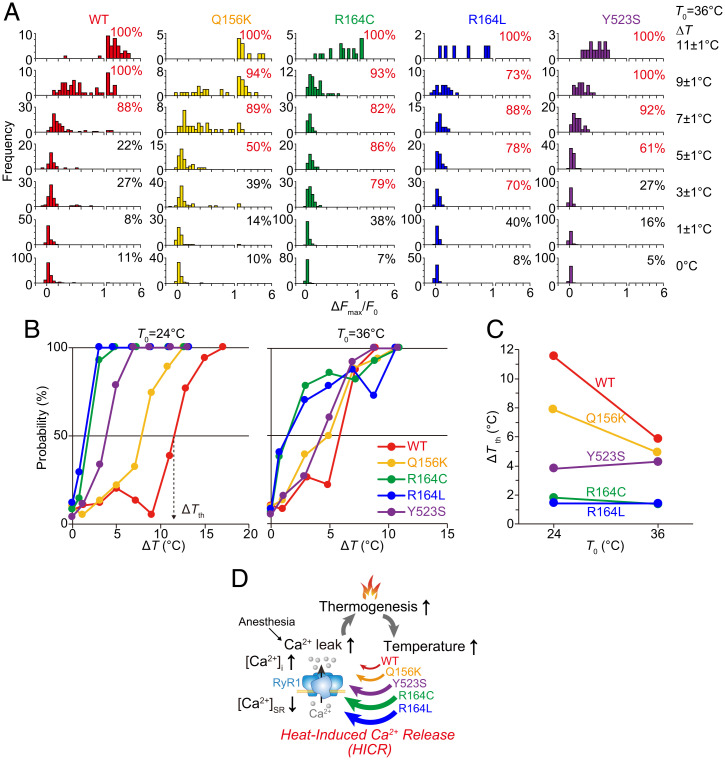
Rank order of RyR1 mutants for heat sensitivity. (*A*) Histograms showing increases in [Ca^2+^]_i_ (Δ*F*_max_/*F*_0_ of fluo-4) in response to heat pulses of various amplitudes in Δ*T*. The number at the upper right of row in each panel indicates the response probability of cells showing significant increases in [Ca^2+^]_i_ (Δ*F*_max_/*F*_0_>Δ*F*_th_). Data in *SI Appendix*, Fig. S6 were analyzed and plotted. *T*_0_ = 36 °C. (*B*) Relationship between Δ*T* and the response probability. The response probability reached 50% at Δ*T*_th_. (*C*) Relationship between *T*_0_ and Δ*T*_th_. Rank order in Δ*T*_th_ at *T*_0_ = 36 °C was R164C (1.4 °C) = R164L (1.4 °C) < Y523S (4.3 °C) < Q156K (4.9 °C) < WT (5.8 °C). The rank order at *T*_0_ = 24 °C was R164L (1.4 °C) ∼R164C (1.8 °C) < Y523S (3.8 °C) < Q156K (7.9 °C) < WT (11.5 °C). (*D*) Schematic illustration of proposed positive feedback loop closed by HICR showing MH initiation and progression of the disease. Anesthesia-triggered Ca^2+^ leak through MH RyR1 mutants induces hyperthermia. The temperature rise destabilizes RyR1 and causes HICR.

Then, the heat sensitivities of WT and RyR1 mutants were examined systematically by exposing cells to heat pulses with varying magnitudes of Δ*T*, as well as by evaluating the fraction of cells exhibiting Ca^2+^ bursts (Fig. 5*A* and *SI Appendix*, Fig. S6). At *T*_0_ = 36 °C, the Ca^2+^ burst was more frequently observed when Δ*T* was larger in WT cells and in all mutant cells. However, the heat sensitivities varied between them. For example, ∼40% of R164C cells showed Ca^2+^ bursts in response to a heat pulse of Δ*T* = 1 °C, but only ∼20% of WT and other mutant cells showed such Ca^2+^ bursts (i.e., R164C cells were most heat sensitive, Y523S and Q156K cells less heat sensitive, and WT cells least heat sensitive). This variation in heat sensitivity was quantitatively compared by using the threshold values of Δ*T* (Δ*T*_th_) at which 50% of cells responded to heating (Fig. 5*B*). This analysis is independent from spontaneous (nonthermal) [Ca^2+^]_i_ fluctuations or the amplitude of the Ca^2+^ burst, either of which can depend on [Ca^2+^]_ER_. Based on this data, we determined the rank order in Δ*T*_th_ as R164C ≪ Y523S < Q156K < WT at both *T*_0_ = 24 °C and 36 °C (Fig. 5 *B*–*D* and *SI Appendix*, Figs. S7 and S8). We further examined R164L, which had a rank order of activity similar to that of R164C (16, 17), and confirmed that the Δ*T*_th_ of R164L was comparable to that of R164C.

### Heat-Induced Ca^2+^ Bursts in Skeletal Muscles from MH-Model Mice.

**Fig. 6.**
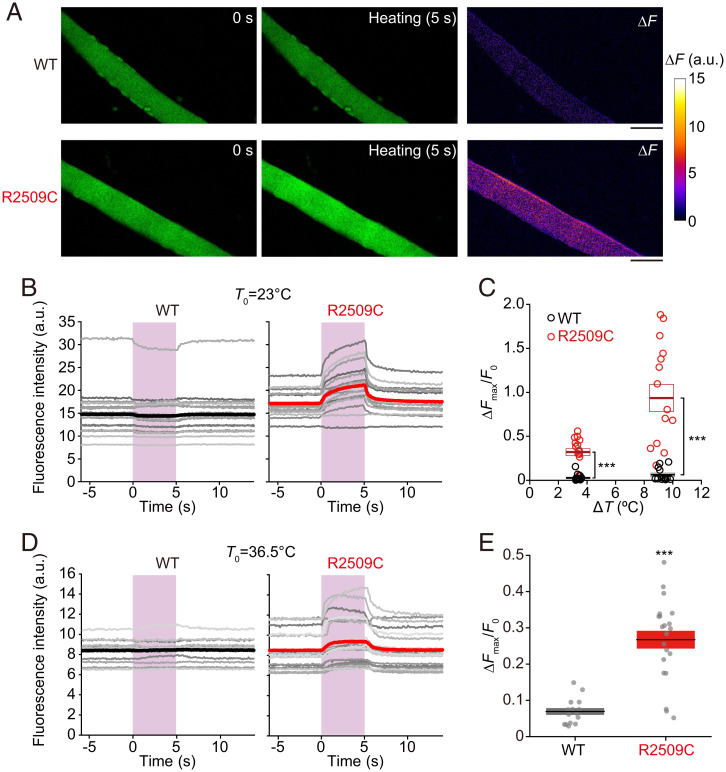
Heat-induced Ca^2+^ bursts in skeletal muscles expressing RyR1 mutant R2509C. (*A*) Confocal images of Cal-520–loaded muscles isolated from WT (*Top*) or R2509C (*Bottom*) mice before (*Left*; *t* = 0 s) and during heating (*Middle*; *t* = 5 s). The microscope and Ca^2+^ indicator employed in this experiment were different from those used for HEK293 cells except for R2508C (*SI Appendix, Materials and Methods*). The right-most figures indicate the differences in fluorescence intensity between *t* = 0 and 5 s (Δ*F*). Δ*T* = 3.5 °C, *T*_0_ = 23 °C. Scale bars, 50 µm. (*B*) Time course of the fluorescence intensity of Cal-520–loaded muscles isolated from WT (*Left*) or R2509C (*Right*) mice. Each gray line represents an individual cell. Thick, colored lines indicate average intensities. Pink vertical bars indicate the periods of the heat pulses. Δ*T* = 3.5 ± 0.5 °C, *T*_0_ = 23 °C. (*C*) Maximum changes in relative fluorescence intensity of Cal-520 Δ*F*_max_/*F*_0_ after the onset of heating. Horizontal bars and boxes indicate means ± SEM. Statistical significance was determined by comparison with WT cells, using the Mann–Whitney *U* test. ***P < 0.001. WT, *n* = 18; R2509C, *n* = 16; and *P* = 6.2 × 10^−7^ for Δ*T* = 3.5 ± 0.5 °C, *T*_0_ = 23 °C. WT, *n* = 13; R2509C, *n* = 15; and *P* = 1.5 × 10^−5^ for Δ*T* = 9 ± 1 °C, *T*_0_ = 23 °C. (*D*) Time course of the fluorescence intensity of Cal-520-loaded muscles isolated from WT (*Left*) or R2509C (*Right*) mice at *T*_0_ = 36.5 °C. Δ*T* = 4.0 ± 0.5 °C. (*E*) Maximum changes in the relative fluorescence intensity of Cal-520 Δ*F*_max_/*F*_0_ after the onset of heating. *T*_0_ = 36.5 °C; Δ*T* = 4.0 ± 0.5 °C. Horizontal bars and boxes indicate means ± SEM. Statistical significance was determined by comparison with WT cells, using the Mann–Whitney *U* test. ***P < 0.001. WT, *n* = 15; R2509C, *n* = 21; and *P* = 1.2 × 10^−6^. a.u., arbitrary units.

Experiments with HEK293 cells demonstrated that Ca^2+^ bursts occurred during heating in cells expressing the RyR1 mutants related to MH. Finally, therefore, we investigated whether the heat-induced Ca^2+^ burst occurs in skeletal muscles from novel MH heterozygous mice expressing RyR1 mutant R2509C (34). In these mice, the rectal temperature increases to >42 °C when exposed to anesthesia (isoflurane) or environmental heat stress (34). Flexor digitorum brevis muscle fibers ∼500 µm long were isolated from WT or R2509C mice (*SI Appendix*, Fig. S9*A*). Resting [Ca^2+^]_i_ was higher in R2509C cells than in WT cells (*SI Appendix*, Fig. S9*B*), which is consistent with the increased resting leak of Ca^2+^ and the consequent enhancement of store-operated Ca^2+^ entry (35), in R2509C compared with WT cells (34, 36). When heat pulses of Δ*T = *3.5 ± 0.5 °C were applied at the base temperature *T*_0_ = 23 °C, there were no detectable [Ca^2+^]_i_ changes during heating in WT muscles (Fig. 6 *A* and *B* and Movie S6). But the [Ca^2+^]_i_ increases were observed in R2509C muscles during heating (Fig. 6 *A* and *B* and Movie S7). An increase in the amplitude of heating to Δ*T =* 9 ± 1 °C elevated [Ca^2+^]_i_ by a slight magnitude in WT muscles (Fig. 6*C* and *SI Appendix*, Fig. S9 *C* and *D* and Movie S8) but caused significantly larger Ca^2+^ bursts in R2509C muscles (Fig. 6*C* and *SI Appendix*, Fig. S9 *C* and *D* and Movie S9). The heat-induced Ca^2+^ bursts were likewise observed in HEK293 cells expressing rabbit RyR1-mutant R2508C, which corresponds to mouse R2509C (*SI Appendix*, Fig. S10). At the physiologically relevant temperature of 36.5 °C, heat-induced [Ca^2+^]_i_ increases were likewise observed in R2509C muscles upon Δ*T =* 4.0 ± 0.5 °C (Fig. 6 *D* and *E*). These findings indicate that [Ca^2+^]_i_ increases dramatically in skeletal muscles of living animals with the RyR1 mutant in response to only an increase in body temperature.

## Discussion

MH is a life-threatening disorder caused by mutations in RyR1, manifested as a sudden and irrepressible body temperature elevation. In the present study, we directly demonstrated that Ca^2+^ was released through RyR1 mutants related to MH upon irradiation of an IR laser beam, and the RyR1 mutants were more heat-sensitive than WT RyR1 cells. The advantages of the present experimental approach are three-fold: First, in the single cell–based assay system, the Ca^2+^-induced Ca^2+^ release (37) through RyR1 mutants can directly be quantified without taking into consideration myopathy development (15–19, 36, 38–44). Second, the expression levels of endogenous RyRs in HEK293 cells can be considered negligible compared with those of the expressed RyR1s (16, 45). Third, transient heating by local heat pulses allows us to avoid the following artifacts: 1) changes in cell morphology; 2) drifting focus due to thermal expansion of components in the experimental set-up; 3) photo-bleaching of fluorophores; 4) relocation of Ca^2+^ indicators due to leakage from, or internalization by intracellular compartments; and 5) thermal damage to biomolecules and cells caused by long periods of heat exposure.

Here, we discuss the possible effects of the local heat pulse on the Ca^2+^ indicators used in the present study. It has been reported that in the range of 20 to 37 °C, a rise in temperature lowers the dissociation constant (*K*_d_) of fluo-4 for Ca^2+^ (*K*_d_ = 520 and 190 nM at 22 and 37 °C, respectively), as well as the fluorescence intensity, due to thermal quenching (maximal currents measured by a photomultiplier *F*_max_ = 1530 and 1300 nA at 22 and 37 °C, respectively) (46). Because the resting [Ca^2+^]_i_ in HEK293 cells expressing RyR1 mutants is within the range of 40 to 90 nM at room temperature (23 to 25 °C) (16), it is likely that the increase in the fluorescence intensity of fluo-4 during heating was coupled with a decrease in *K*_d_ independent of a heat-induced increase in [Ca^2+^]_i_. Even if this is the case, the temperature-dependent property of fluo-4 would not affect the response probability in HEK293 cells expressing WT, Q156K, or Y523S RyR1 (Fig. 5 and *SI Appendix*, Fig. S8). This is because an increase in the fluorescence intensity of fluo-4 was observed after the cessation of heating in these cells (Fig. 2 and *SI Appendix*, Figs. S6 and S7). However, Δ*F*_max_/*F*_0_ of fluo-4 could be reached during heating in cells expressing R164C or R164L. Then, the response probability may have been either overestimated or underestimated in these cells. The elevation of the fluorescence intensity of fluo-4 was insignificant because the signal was not sufficiently increased in cells expressing WT, Q156K, and Y523S during heating (Fig. 2*E*). If a substantial signal decrease occurred during heating, the response probability would have been underestimated. Namely, the Δ*T*_th_ values determined in R164C and R164L cells could have been overestimated (Fig. 5 *A* and *B* and *SI Appendix*, Fig. S8). In other words, the Δ*T*_th_ values of these cells could have been smaller than those obtained in the present study and, hence, substantially more heat hypersensitive. In this case, the rank order of Δ*T*_th_ (Fig. 5*C*) will remain unchanged, although it is, at present, difficult to quantitatively examine the magnitude of overestimation of Δ*T*_th_. We employed G-CEPIA1*er* as another fluorescence Ca^2+^ indicator in the present study (Fig. 4*B*). During measurement, the G-CEPIA1*er* fluorescence intensity after heating was analyzed; it was not affected by either the temperature sensitivity of the Ca^2+^ affinity or the thermal quenching of G-CEPIA1*er*. We would like to stress the positive correlation between the amplitude of [Ca^2+^]_ER_ decrease (−Δ*F*_min_/*F*_0_ of G-CEPIA1*er*) and that of the Ca^2+^ burst (Δ*F*_max_/*F*_0_ of fluo-4) (Fig. 4*C*). In skeletal muscles, contraction was clearly observed in response to an increase in [Ca^2+^]_i_ during heating (*SI Appendix*, Fig. S9 and Movie S9). These findings clearly demonstrate that heat-induced Ca^2+^ bursts are not the temperature-dependent properties of fluorescent Ca^2+^ indicators.

The heat hypersensitivity of RyR1 mutants suggests that a positive feedback between Ca^2+^ release and thermogenesis accelerates the progression of MH. It has been reported that metabolic activity is higher in R163C mice than in WT mice (47). In a single-fiber assay, it has been demonstrated that the thermogenesis by SERCA is closely coupled with Ca^2+^ leakage through RyR1 under resting conditions (48). It is accordingly suggested that when the Ca^2+^ leakage through RyR1 mutants is enhanced by MH inducers (e.g., inhalation of anesthetics), the resultant increase in [Ca^2+^]_i_ exacerbates the SERCA-based thermogenesis. The present study documented that an increase in ambient temperature of <2 °C causes Ca^2+^ release through the channels (Fig. 5). We designate this mechanism as heat-induced Ca^2+^ release (HICR). HICR was observed in WT cells and all RyR1 mutant cells as Ca^2+^ bursts during heating with a relatively strong pulse (see data for Δ*T =* 11 ± 1 °C in *SI Appendix*, Fig. S6). With a weaker pulse, HICR was somewhat unclear during heating; rather, it appeared as sustained Ca^2+^ bursts after recooling. We previously reported that prolonged Ca^2+^ bursts occur upon recooling in HeLa (21) and WI-38 (11) cells, with a mechanism similar to that of rapid cooling contractures (49, 50). It is therefore likely that the recooling-induced Ca^2+^ burst is participating in the increased [Ca^2+^]_i_ after recooling. Ca^2+^ leak properties of Y523S, as compared with other channels, may underlie the lesser magnitude of Ca^2+^ bursts (as discussed below). Both SERCA and RyR1 are localized in the ER membrane; it is therefore possible that HICR plays a critical role in the progression of MH. We previously examined the increase in rectal temperature induced by isoflurane in R2509C mice and stated that the elevation of the rectal temperature appeared as a two-phase reaction: it rose slowly at first and then surged at ∼39 °C (34). This finding is consistent with the notion that the positive feedback loop operates in malignant hyperthermia in vivo, which is coupled, at least in part, with HICR. The heat transiently released during HICR was too small to be detected using luminescence thermometry at the SR (51, 52) in individual skeletal muscle fibers (*SI Appendix*, Fig. S11). In future studies, therefore, rapid heating combined with simultaneous imaging of Ca^2+^ and temperature should be performed in skeletal muscles of living mice to quantitatively analyze the contribution of HICR in the progression of malignant hyperthermia.

We consider that HICR coincides with the positive feedback loop in a relatively long time scale that Durham et al. (9) and Lanner et al. (53) suggested. They showed that increased [Ca^2+^]_i_ activates the production of both reactive oxygen species (ROS) and reactive nitrogen species (RNS) in heterozygous knock-in mice with a Y524S mutation (Y522S in humans) for a longer period of time compared with the present study. Either ROS or RNS, or both may modify RyR1 and other skeletal muscle proteins to promote Ca^2+^ release via unknown mechanisms. Because of the relatively short time scale of HICR, the slower processes of ROS and RNS production and posttranscriptional modifications may enhance HICR and thereby aggravate MH.

We found that Δ*T*_th_ is smaller in R164C and Y523S cells than in WT cells (Fig. 5). This is consistent with previous findings by others: 1) muscle contraction and sudden death can be triggered by merely a moderate temperature rise in R165C (R163C in humans) and Y524S knock-in mice [corresponding to rabbit R164C and Y523S mutants, respectively (6, 7)], and 2) halothane-induced increases in [Ca^2+^]_i_ are accelerated in muscle fibers from Y524S knock-in mice upon moderate heating from 25 to 35 °C (54). We also found that the rank order of activity did not necessarily reflect the order of heat sensitivity. For example, while the Y523S channel mutant was leakier than R164C(L) (Fig. 1*A* and *SI Appendix*, Figs. S12 and S13 and *SI Appendix, Supplemental Results and Discussion*) (16, 18, 19), Δ*T*_th_ was greater in Y523S cells than in R164C(L) cells (Fig. 5*C*). Because the Y523S mutant is the most destabilized channel, more Ca^2+^ is released even without heating. Thus, the depletion of [Ca^2+^]_ER_ of Y523S cells could be particularly crucial in limiting the magnitude of Ca^2+^ bursts via HICR. Accordingly, the Ca^2+^ bursts may be more pronounced in Q156K and R164C cells under conditions where [Ca^2+^]_ER_ is less depleted. Therefore, in order to investigate the effect of the [Ca^2+^]_ER_ depletion on Ca^2+^ bursts, we normalized the magnitude of the Ca^2+^ bursts (i.e., Δ*F*_max_/*F*_0_) (Fig. 2) by the relative resting [Ca^2+^]_ER_ of each mutant to that of WT (*SI Appendix*, Fig. S14). This analysis revealed strong responses in R164C and Y523S cells, suggesting that the Δ*F*_max_/*F*_0_ values in Fig. 2 underestimate the magnitude of HICR when resting [Ca^2+^]_ER_ is low (*SI Appendix*, Fig. S13). These data support the relevance of the comparison of heat sensitivity between mutants as determined on the basis of response probability (Fig. 5).

The levels of resting [Ca^2+^]_i_ and [Ca^2+^]_ER_ in mutant cells were significantly higher and lower, respectively, than those in −Dox and WT cells (*SI Appendix*, Figs. S12 and S13). Because the SERCA activity depends on [Ca^2+^] in both the cytosol and ER (55), this activity is more pronounced in the mutant cells than in −Dox or WT cells. This higher activity of SERCA in the mutant cells can be further accelerated by a temperature rise upon heating, resulting in a marked [Ca^2+^]_i_ decrease during heating. This interpretation is consistent with the response of some Q156K cells that exhibited remarkably higher fluo-4 intensities than the average due to spontaneous Ca^2+^ oscillations before heating. These cells had marked decreases in [Ca^2+^]_i_ during heating [e.g., see Q156K cells whose fluorescence intensity values were at ∼70 arbitrary units before heating at 24 °C (Fig. 2*C*) and 36 °C (Fig. 2*E*)]. This phenomenon could not simply be explained by thermal quenching of fluo-4, because the decreased [Ca^2+^]_i_ did not return to its original level after the cessation of heating. We consider that the heat-enhanced SERCA activity likewise occurred in R164C and R164L cells. However, the [Ca^2+^]_i_ increased during heating (Fig. 2*C*). This result can be interpreted as follows: The Ca^2+^ release via HICR is more pronounced than the heat-enhanced Ca^2+^ uptake by SERCA in these mutants, hence the net Ca^2+^ flux occurs from the ER lumen to the cytosol. This interpretation is consistent with the higher heat sensitivity observed in these mutants (Fig. 5). The higher resting [Ca^2+^]_i_ in mutant cells can, at least in part, account for the differences in the magnitude of the Ca^2+^ decline after heating among mutant cell lines (Fig. 2). The Ca^2+^ decline can be faster in the leakier R164C and Y523S cells than in WT cells, as observed in Fig. 2*E*. Considering these two mutants, the Ca^2+^ decline is considered to be faster in R164C cells than in Y523S cells.

We consider that the heat hypersensitivity of RyR1 mutants related to MH is caused by destabilized interactions between the N-terminal domains (A, B, and C) and the neighboring domains at the N-terminal “hotspot” of residues 35 to 614, one of three known hotspots related to MH in RyR1 (3, 56) (*SI Appendix*, Fig. S15). The Q156 and R164 in domain A interact with domain B to form interface 1 within the ABC subunit at the N terminus (57). At the bottom of domain A (interface 4), R164 interacts with the core domain (57). Y523 is exposed at the surface of domain C, facing the cytosolic shell domain (interface 3) (57). One plausible explanation for the present data is that the interdomain interactions are more easily destabilized by heat in the mutants than in the WT, resulting in Ca^2+^ leakage and subsequent intracellular Ca^2+^ bursts. This idea is supported by previous studies, namely 1) RyR1 mutant structures are unstable under environmental heat stress (57–59) and 2) ER Ca^2+^ levels in R164C(L) and other N-terminal mutant cells relative to WT cells are lower at 36 °C than at room temperature (16). It is, therefore, reasonable to conclude that the heat hypersensitivity observed in the present study is a fundamental property for RyR1 mutants related to MH at the N-terminal hotspot. We demonstrated that HICR occurred in R2509C skeletal muscle cells (Fig. 6). Therefore, the mutants in other hotspots containing R2509C may likewise be heat hypersensitive via a similar mechanism. Future studies employing molecular dynamics simulations (58, 60, 61) are warranted to investigate the effects of interdomain interface mutations on the heat sensitivity of RyRs. The HICR in R2508C HEK293 cells (*SI Appendix*, Fig. S10) was observed in a fashion different from that in muscle cells (Fig. 6 and *SI Appendix*, Fig. S9). Because homozygous mice are lethal (34), skeletal muscles were obtained from heterozygous R2509C mice in the present study. Therefore, we consider that a greater amount of Ca^2+^ leak, hence a higher resting [Ca^2+^]_i_ coupled with a lower resting [Ca^2+^]_ER_, is demonstrated in R2508C HEK293 cells, compared with that in muscles isolated from R2509C mice. Accordingly, HICR occurs in R2508C HEK293 cells, but the magnitude is less than that observed in skeletal muscle cells from R2509C mice. It should be pointed out that, in skeletal muscles, RyR1 interacts with other modulators such as dihydropyridine receptors in a temperature-dependent manner (62). Therefore, in future studies using various mutants, systematic experimental approaches should be implemented to clarify the complexity of the molecular mechanisms of HICR in vivo.

In summary, by taking advantage of local heating technology, we demonstrated that abnormal RyR1 heat sensing caused HICR in RyR1 mutant–expressing HEK293 cells and in skeletal muscles from MH mice. These findings suggest that an additional positive feedback loop between thermogenesis and heat-sensing via heat-hypersensitive RyR1 mutants irrepressibly elevates body temperature during MH and, presumably, during exertional heat stroke under extreme environmental conditions (63–65).

## Materials and Methods

### Chemicals.

Dulbecco's Modified Eagle Medium (DMEM) (catalog no. 08488-55), l-glutamine (catalog no. 16948-04), and hygromycin (catalog no. 09287-84) were purchased from Nacalai Tesque Inc. (Kyoto, Japan). Fetal bovine serum (FBS; catalog no. 10437-028), penicillin and streptomycin (catalog no. 15140-122), Lipofectamine 2000 (catalog no. 11668), and fluo-4 AM (catalog no. F14217) were purchased from Thermo Fisher Scientific (Waltham, MA). Blasticidin (ant-bl-1) was purchased from InvivoGen (San Diego, CA). Collagen type 1 (catalog no. IFP9660) was purchased from the Research Institute for the Functional Peptides (Yamagata, Japan). Dox (catalog no. D9891), 2-APB (catalog no. D9754), and poly (methyl methacrylate) (PMMA; molecular weight [M_w_] ∼15,000) (catalog no. 200336) were purchased from Sigma-Aldrich (St. Louis, MO). Thapsigargin (catalog no. 586005) was purchased from Merck (Darmstadt, Germany). Ryanodine (catalog no. 185-02821) was purchased from FUJIFILM Wako Pure Chemical Corporation (Osaka, Japan). Europium (III) thenoyltrifluoroacetonate trihydrate (Eu-TTA) (catalog no. 21392-96-1) was purchased from Acros Organics (Pittsburgh, PA). The stocks of 1 mM fluo-4 AM in dimethyl sulfoxide (DMSO), 2 mM thapsigargin in DMSO, 10 mM ryanodine in distilled water, and 100 mM 2-APB in DMSO were stored at −20 °C until use.

### Cell Culture.

HEK293 cells stably transformed with rabbit skeletal muscle RyR1 or its mutants (Q156K, R164C(L), Y523S, or R2508C; human Q155K, R163C(L), Y522S, or R2508C) were generated, and the expression levels of WT RyR1 and the RyR1 mutants were confirmed to be similar in previous studies (15–17, 66). The expression of RyR1 is inducible by Dox using the Flp-In T-Rex system (Thermo Fisher Scientific); hence, the system is suitable to investigate the functions of fatal RyR1 mutants in living cells.

The HEK293 cells were cultured in flasks or on dishes coated with collagen (TPP Techno Plastic Products AG, Trasadingen, Switzerland) in culture medium (DMEM containing 10% FBS, 2 mM l-glutamine, 100 units/mL penicillin, and 100 μg/mL streptomycin) with 100 μg/mL hygromycin and 15 μg/mL blasticidin at 37 °C in 5% CO_2_. To coat the flasks and dishes with collagen, they were filled with a 0.001% collagen type 1 solution in distilled water for 1 h at 37 °C. The flasks and dishes were washed with the fresh culture medium just before use.

### Ca^2+^ Imaging.

Cells were seeded on collagen-coated glass base dishes (3911-035; AGC Techno Glass, Shizuoka, Japan) at 37 °C in 5% CO_2_ for 1 to 3 d. To induce RyR1 expression, the culture medium was replaced with the medium containing 2 μg/mL Dox. Cells were incubated for 24 to 36 h before experiments.

Cytoplasmic Ca^2+^ dynamics were studied using the fluorescent Ca^2+^ probe fluo-4 AM. The cells were incubated in HEPES-buffered saline (HBS; 140 mM NaCl, 5 mM KCl, 1 mM MgCl_2_, 1 mM Na_2_HPO_4_, 10 mM HEPES, 2 mM CaCl_2_, 5 mM d-glucose, pH 7.4, adjusted with NaOH) containing 1 µM fluo-4 AM for 30 min at room temperature. The solution was replaced with fresh HBS, and the cells were incubated under the microscope for at least 10 min before observation to allow temperature stabilization to 24 ± 1 °C or 36 ± 0.5 °C. In some experiments, the cells were observed in Ca^2+^-free solution (140 mM NaCl, 5 mM KCl, 1 mM MgCl_2_, 1 mM Na_2_HPO_4_, 10 mM HEPES, 5 mM d-glucose, 2 mM ethylene glycol tetraacetic acid, pH 7.4, adjusted with NaOH) at least 15 min after incubation. To inhibit the activity of SERCA or IP_3_R, the cells were incubated in HBS containing 1 µM fluo-4 AM and either 2 µM thapsigargin or 100 µM 2-APB for 30 min at 24 °C, and then observed in HBS containing 2 µM thapsigargin or 100 µM 2-APB. To inhibit RyR1, the fluo-4–loaded cells were observed in HBS containing 100 µM ryanodine.

To image Ca^2+^ dynamics in the ER, HEK293 cells stably expressing G-CEPIA1*er* (33) were prepared as follows: lentiviral vectors harboring the G-CEPIA1*er* construct were produced by replacing the complementary DNA of enhanced green fluorescent protein (GFP) in pCL20c-MSCV-AcGFP-WPRE (kindly provided by Dr. Y. Ohashi, The University of Tokyo, Tokyo, Japan) (67). HEK293T cells were cotransfected with four plasmids—pCAGkGP1.1R, pCAG4RTR2, pCAG-VSV-G, and pCL20c-MSCV-G-CEPIA1er-WPRE—using Lipofectamine 2000. The cells were incubated at 37 °C in 5% CO_2_ for 16 h. Then the culture medium was replaced, and the cells were incubated at 37 °C in 5% CO_2_ for 36 h. The lentivirus-containing medium was collected and cleared by centrifugation at 1,500 rpm for 5 min at 4 °C. To concentrate the lentivirus, the supernatant was centrifuged at 10,000 rpm at 4 °C overnight. The pellets were suspended in 50 μL of phosphate-buffered saline and stored at −80 °C until use. HEK293 cells expressing WT RyR1 or the RyR1 mutants were transfected with the virus for G-CEPIA1*er*. The cells of ∼80 to 95% were transduced. After at least two additional passages, the cells were used in the experiments.

### Optical Set-up.

The microscope with the local heating system was described in detail in our previous reports (10–14, 20). Briefly, the local temperature around the cell was increased by a 1,455-nm IR laser beam that is efficiently absorbed by water (KPS-STD-BT-RFL-1455–02-CO; Keopsys, Lannion, France). The duration of irradiation was controlled by a mechanical shutter (SSH-C4B; SIGMAKOKI, Tokyo, Japan). The laser power was measured using a thermal disk sensor (LM-3; Coherent, Santa Clara, CA) and a power meter (FieldMaster; Coherent) at the level of the sample after passage through the objective lens. The permeability of the IR laser beam, which is the ratio of the output laser power, at the level of the sample, to the input laser power, was ∼1.6%. Fluo-4, G-CEPIA1*er*, and Eu-TTA were excited by a solid-state illuminator (SPECTRA Light Engine; Lumencor, Beaverton, OR; 377/50 nm for Eu-TTA and 485/20 nm for fluo-4 and G-CEPIA1*er*). The fluorescence and the bright-field images were observed with an inverted microscope (IX70; Olympus, Tokyo, Japan) equipped with a dichroic mirror (DM505; Olympus), an emission filter (BA515IF; Olympus), an objective lens (PlanApo N 60×/1.45 oil; Olympus), and an electron-multiplying charge-coupled device camera (iXon^EM^ + 897; Andor Technology, Belfast, UK). The temperature of the solutions on the sample stage was adjusted to 36 ± 0.5 °C using a thermostatically controlled incubator (INUCP-KRi-H2-F1; Tokai Hit, Shizuoka, Japan); otherwise, the temperature of the solutions was 24 ± 1 °C.

### Analyses.

The microscopic images were analyzed with the ImageJ software (NIH, Bethesda, MD). The changes in local temperature were measured by thermal quenching of Eu-TTA that had been spin-coated on a glass base dish by a solution containing 5 mg/mL Eu-TTA and 10 mg/mL PMMA in acetone (11–13). Relative changes in the intensity of Eu-TTA were calculated by Δ*F*/*F*_0_ = (*F*_heating_ − *I*_laser_)/(*F*_before_ − *I*_back_) − 1, where *F*_heating_ was the intensity at the end of the heating period (i.e., just before the IR laser beam was shut off), and *I*_laser_ was the background intensity caused by light scattering of the IR laser beam. *I*_back_ was the background intensity when the excitation light was off. Photo-bleaching was corrected by fitting a single exponential curve. Δ*F*/*F*_0_ of Eu-TTA was converted to Δ*T* using the relationship between Δ*T* and Δ*F*/*F*_0_ (−2.7% °C^−1^ at 24 °C and −4.1% °C^−1^ at 36 °C) (11, 13).

To calculate changes in the fluorescence intensities of fluo-4 and G-CEPIA1*er*, the outlines of cells were manually tracked in the bright-field images. Then the fluorescence intensities of fluo-4 and G-CEPIA1*er* were measured within the outlined areas. The distance between the area center and a laser spot was defined as the distance between the cell and the heat source. The Δ*F* of fluo-4 was calculated from *F* − *F*_before_, where *F* was the fluorescence intensity at an arbitrary time, and *F*_before_ was the intensity just before heating was initiated (i.e., 10 s after beginning the observation). The basal fluorescence intensity of fluo-4 (*F*_0_) was calculated from *F*_before_ − *I*_back_. The peak intensity of fluo-4 (Δ*F*_max_/*F*_0_) was calculated from the maximum Δ*F*/*F*_0_ during the 20 s after heating initiation. Light scattering by the IR laser beam (Movie S1) was subtracted to calculate Δ*F*_max_/*F*_0_ during heating. No noticeable photo-bleaching of fluo-4 was observed during the measurement (Fig. 2*C*).

The Δ*F*_max_/*F*_0_ of spontaneous [Ca^2+^]_i_ fluctuations in *SI Appendix*, Fig. S16 was calculated using (*F*_max_ − *F*_before_)/(*F*_before_ − *I*_back_). To equalize the exposure time of excitation light (485 nm) and that in the heating experiments, *F*_before_ was set to the fluorescence intensity 10 s after starting the observation. *F*_max_ was the maximum intensity obtained from 10 to 30 s after starting the observation. The cumulative probability of Δ*F*_max_/*F*_0_ was fitted by a cumulative distribution function of the Gaussian distribution (68), as follows:

$$F\left(x\right)=\;\frac{1}{\sqrt{2\sigma^{2}\pi }}{\int_{-\infty }^{x}\text{e}}^{-\frac{{\left(t-\mu \right)}^{2}}{2\sigma^{2}}}dt,$$

where µ and σ are the mean and the SD of the Gaussian distribution, respectively. The fitting by least squares methods was performed in Excel 2016 (Microsoft, Redmond, WA) using the following equation:

$$F\left(x\right)=\;\frac{1}{2}\left[1+\text{erf}\left(\frac{x-\mu }{\sqrt{2\sigma^{2}}}\right)\right],$$

where erf(*x*) is an error function. The threshold Δ*F*_th_ of Δ*F*_max_/*F*_0_ was defined as µ + 1.96σ. If Δ*F*_max_/*F*_0_ induced by a heat pulse was higher than Δ*F*_th_, the [Ca^2+^]_i_ increase response induced by the heat pulse was considered significant. The threshold of Δ*T* (Δ*T*_th_) was defined as the Δ*T* that induced a significant [Ca^2+^]_i_ increase (Δ*F*_max_/*F*_0_>Δ*F*_th_) in 50% of cells (Fig. 5*B*).

The Δ*F*_min_/*F*_0_ of G-CEPIA1*er* was calculated from (*F*_min_ − *F*_before_)/(*F*_before_ − *I*_back_), where *F*_min_ was the minimum intensity of G-CEPIA1*er* obtained from 2.4 s to 10 s after heating initiation. In this calculation of Δ*F*_min_/*F*_0_, photo-bleaching was corrected by fitting with a single exponential curve in Excel 2016 (Microsoft).

### Skeletal Muscle Cells.

Animal-related procedures were in accordance with the guidelines of The Jikei University School of Medicine. Mice were housed in isolator cages, fed with food and water ad libitum, and kept under controlled lighting conditions (12 h-light/12 h-dark) in specific pathogen-free conditions at The Jikei University School of Medicine. Isolated skeletal muscle cells from mice were prepared based on a previously described procedure with modifications (34). Briefly, WT and R2509C mice were anesthetized with intraperitoneal injection of an anesthetic mixture (0.75 mg/kg medetomidine, 4 mg/kg midazolam, and 5 mg/kg butorphanol) before euthanasia. Flexor digitorum brevis muscles were dissected and incubated with 2 mg/mL collagenase (Worthington Biochemical, Lakewood, NJ) in the HEPES–Krebs solution (140 mM NaCl, 5 mM KCl, 2 mM CaCl_2_, 1 mM MgCl_2_, 11 mM glucose, 5 mM HEPES, pH 7.4) containing 2 mg/mL bovine serum albumin (A8806; Merck, Darmstadt, Germany) for 2 h at 37 °C, followed by gentle trituration in collagenase-free HEPES–Krebs solution. The procedures for experiments with skeletal muscles are described in *SI Appendix, Supplemental Materials and Methods*.

### Statistical Analysis.

Multiple groups were compared using the Steel or Dunnett’s test. For comparisons of two independent samples, the Mann–Whitney *U* test was used. These tests were performed using EZR (version 1.51) (69). Statistical significance was described by *P* values. Linear regression analysis was performed using OriginPro2021b software (OriginLab, Northampton, MA). The temperature and *ΔT* are reported as central value ± range where applicable.

## Supplementary Material

Supplementary File

Supplementary File

Supplementary File

Supplementary File

Supplementary File

Supplementary File

Supplementary File

Supplementary File

Supplementary File

Supplementary File

## Data Availability

All study data are included in the article and/or *SI Appendix*.
