# Supplementary material for: Heat-hypersensitive mutants of ryanodine receptor type 1 revealed by microscopic heating
Source: Proc Natl Acad Sci U S A. 2022 Aug 4;119(32):e2201286119. doi: 10.1073/pnas.2201286119 (PMC9371657; doi:10.1073/pnas.2201286119)
Supplement: Supplementary File [file pnas.2201286119.sapp.pdf]

Supporting Information

Heat-hypersensitive mutants of ryanodine receptor type 1 revealed by microscopic heating

Kotaro Oyama, Vadim Zeeb, Toshiko Yamazawa, Nagomi Kurebayashi, Fuyu Kobirumaki-Shimozawa, Takashi Murayama, Hideto Oyamada, Satoru Noguchi, Takayoshi Inoue, Yukiko U. Inoue, Ichizo Nishino, Yoshie Harada, Norio Fukuda, Shin'ichi Ishiwata, Madoka Suzuki

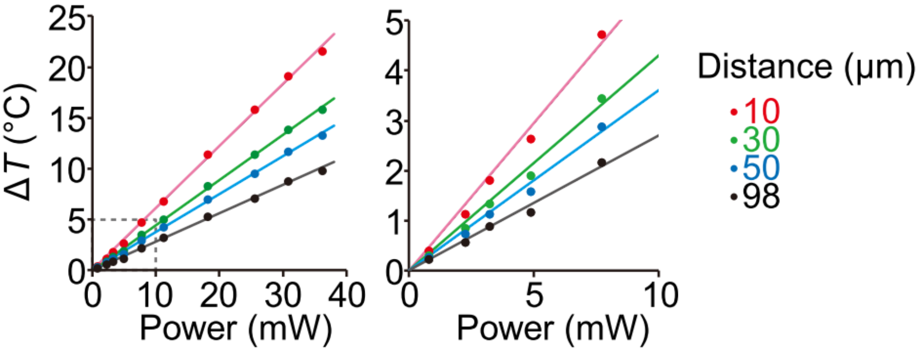

Figure S1. Relationship between laser power and temperature elevation (Δ*T*) at various distances from the heat source. Left, Δ*T* was measured on the surface of a glass base dish by the thermal quenching of the temperature-sensitive dye europium (III) thenoyltrifluoroacetonate trihydrate. Right, enlarged view of the area demarcated by dashed lines in the left panel. Error bars representing standard errors (SEM) (*n*=3 measurements) are within symbols.

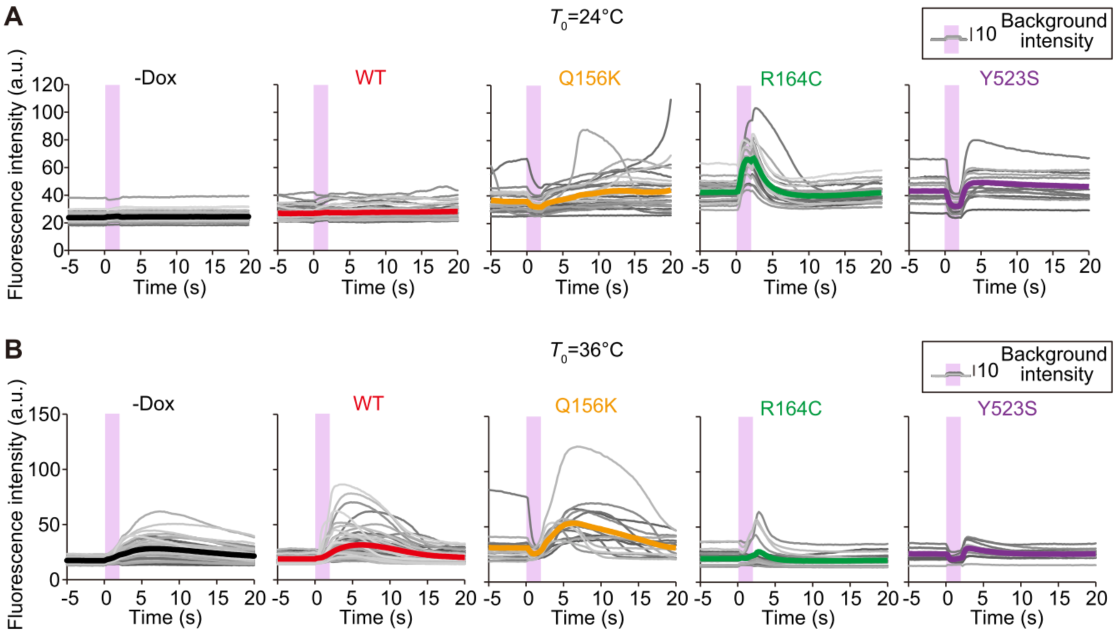

Figure S2. Heat-induced Ca^{2+} bursts in HEK293 cells expressing ryanodine receptor type 1 mutants. (**A**) Changes in the fluorescence intensity of fluo-4 in cells without induction of RyR1 expression [−doxycycline (−Dox)] as a control, or with induction of the expression of WT RyR1, or the mutants (Q156K, R164C or Y523S). Each gray line represents an individual cell. Thick colored lines indicate the average intensities. Pink vertical bars indicate the period of heating. *Inset*, changes in background intensity during heating caused by IR laser beam scattering (n=40). Laser power, 25.6 mW; ΔT=10±2°C; T_0=24°C. (**B**) Changes in the fluorescence intensity of fluo-4 in HEK293 cells at 36°C. Laser power, 25.6 mW; ΔT=10±1°C; T_0=36°C.

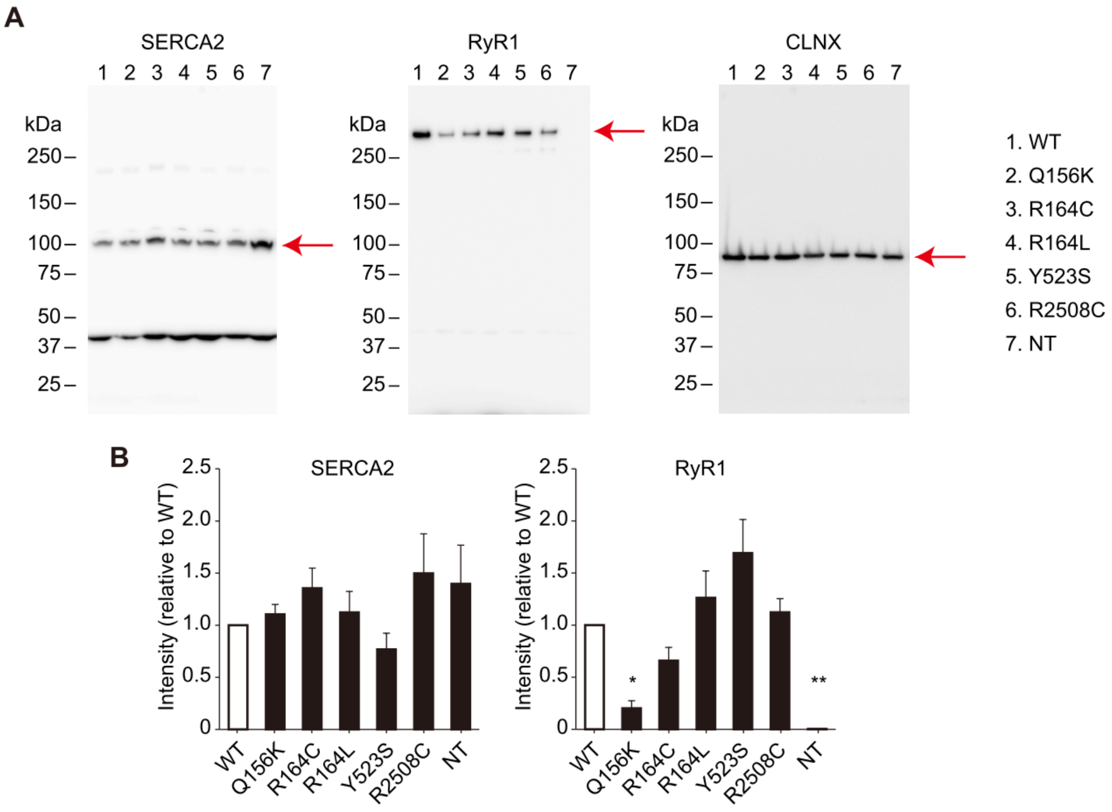

Figure S3. Expression levels of SERCA2 and RyR1 in HEK293 cells. (A) Western blotting showing SERCA2 (left), RyR1 (middle), and loading control Calnexin (CLNX) (right) in HEK293 cells expressing wild-type (WT) or mutant RyR1 (Q156K, R164C, R164L, Y523S, R2508C), or non-transfected (NT) HEK293 cells (i.e., lanes 1–7 as shown on right). Molecular mass standards are shown on the left (kDa). Red arrows indicate targeted proteins. (B) Expression levels of SERCA (left) and RyR1 (right) analyzed from the band intensities. In both graphs, data were normalized by WT. Left: SERCA2 levels were similar between groups (p>0.05). Right: RyR1 level was significantly lower in Q156K or NT cells than in WT cells (p=0.026 for Q156K, $p=5.9 \times 10^{-3}$ for NT, Dunnett's test, *p<0.05, **p<0.01). Means ± SEM (n=3).

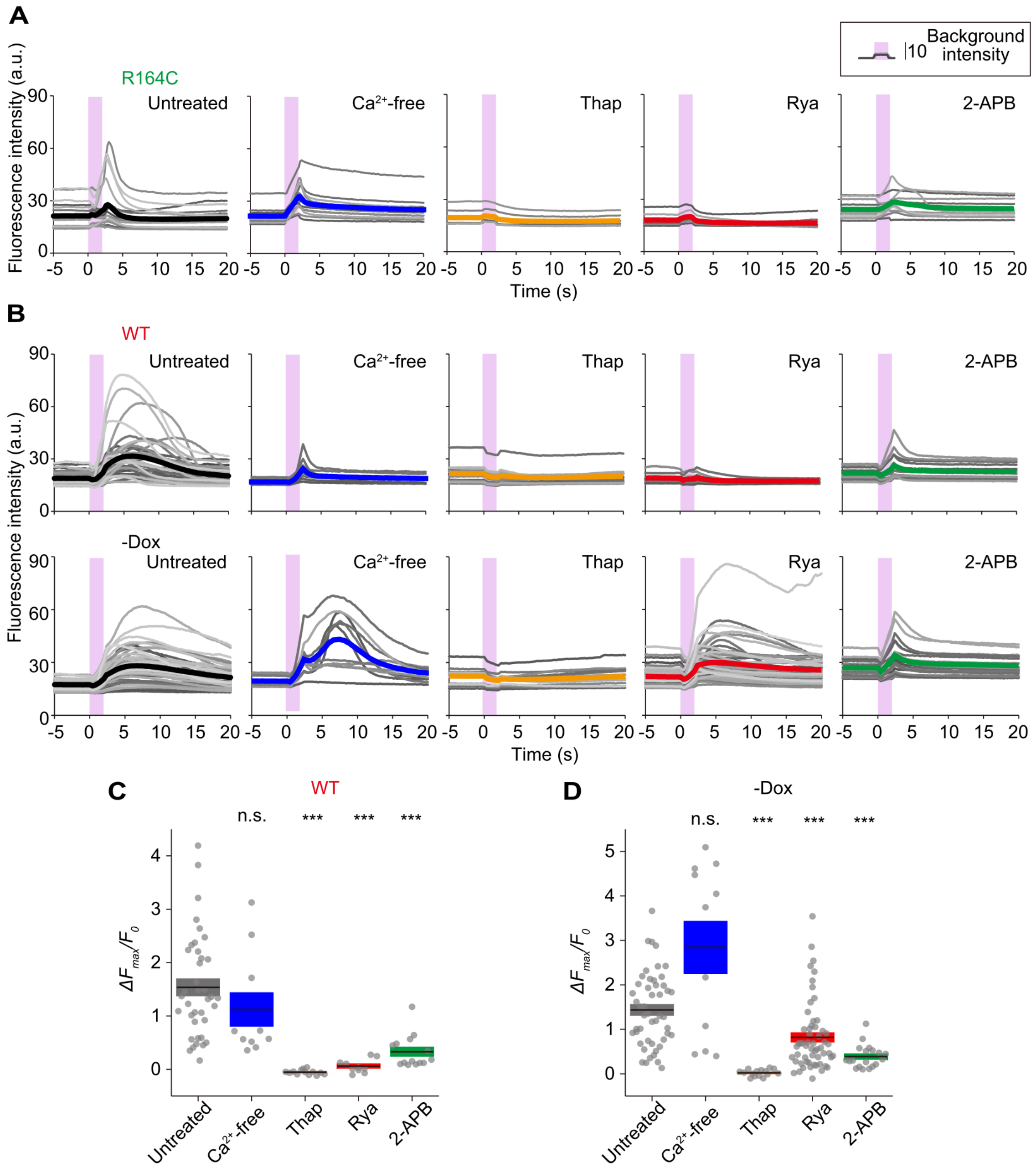

Figure S4. Heat-induced Ca^{2+} bursts in R164C, WT and -Dox cells under various conditions. (**A**) Time courses of the fluorescence intensity of fluo-4 in HEK293 cells expressing R164C in an untreated condition, in Ca^{2+}-free solution (Ca^{2+}-free), and in the presence of 2 µM thapsigargin (Thap), 100 µM ryanodine (Rya), or 100 µM 2-aminoethyl diphenylborinate (2-APB). Each gray line represents an individual cell. Thick colored lines represent averages. Pink vertical bars indicate the period of heating. *Inset*, changes in

the background intensity during heating caused by IR laser beam scattering (n=7). (**B**) Time course of the fluorescence intensity of fluo-4 in HEK293 cells with [wild-type (WT)] or without [−doxycycline (−Dox)] induced WT ryanodine receptor type 1 expression in an untreated condition, in Ca^{2+}-free solution, and in the presence of 2 µM thapsigargin, 100 µM ryanodine, or 100 µM 2-APB. Each gray line represents an individual cell. Changes in the background intensities caused by IR laser beam scattering were subtracted from the fluo-4 signals. Thick colored lines represent averages. Pink vertical bars indicate the period of heating. (Top) WT, (Bottom) −Dox. (**C, D**) Maximum changes in relative fluorescence intensity $\Delta F_{max}/F_0$. Horizontal bars and boxes indicate means ± SEM. (**C**) WT, (**D**) −Dox. Statistical significance was examined by comparison with the untreated cells (WT, n=38; −Dox, n=49) using the Steel test (***p<0.001; n.s., not significant). Data for WT, p=0.51 (Ca^{2+}-free, n=10), 3.9×10^{-7} (Thap, n=13), 7.2×10^{-6} (Rya, n=10), and 1.4×10^{-5} (2-APB, n=14). Data for −Dox, p=0.18 (Ca^{2+}-free, n=11), 2.4×10^{-8} (Thap, n=15), 1.5×10^{-4} (Rya, n=57), and 2.3×10^{-6} (2-APB, n=19). Laser power, 25.6 mW; ΔT=10±1°C; T_0=36°C. Note that despite the lack of a significant difference in maximal amplitudes, the kinetics of the fluo-4 intensity in Ca^{2+}-free solution differed markedly from that in untreated-cells.

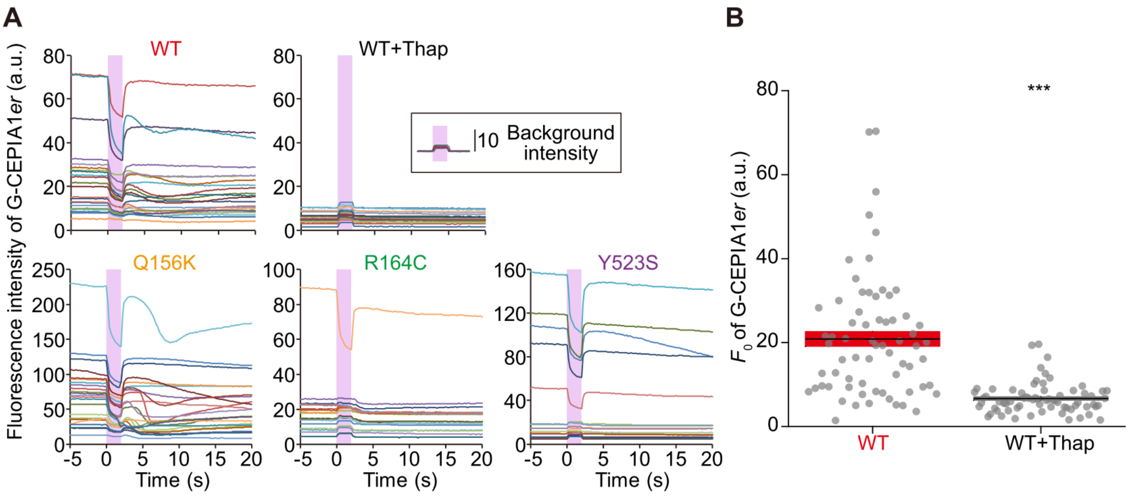

Figure S5. Ca^{2+} dynamics in the endoplasmic reticulum of individual cells. (**A**) Time course of changes in the fluorescence intensity of G-CEPIA1*er* in HEK293 cells expressing wild-type (WT) ryanodine receptor type 1 (RyR1) with or without 2 μM thapsigargin and in cells expressing RyR1 mutants. Pink vertical bars indicate the period of heating . Each line represents an individual cell. *Inset*, changes in the background intensity during heating caused by IR laser beam scattering (n=22). (**B**) G-CEPIA1*er* fluorescence intensity without heating, F_0. For untreated and thapsigargin-treated cells, n=67 and 69 cells, respectively. Horizontal bars and boxes indicate means ± SEM. Statistical significance was examined using the Mann–Whitney *U* test (***p<0.001). $p=6.2 \times 10^{-14}$. Laser power, 25.6 mW; ΔT=10±1°C; T_0=36°C.

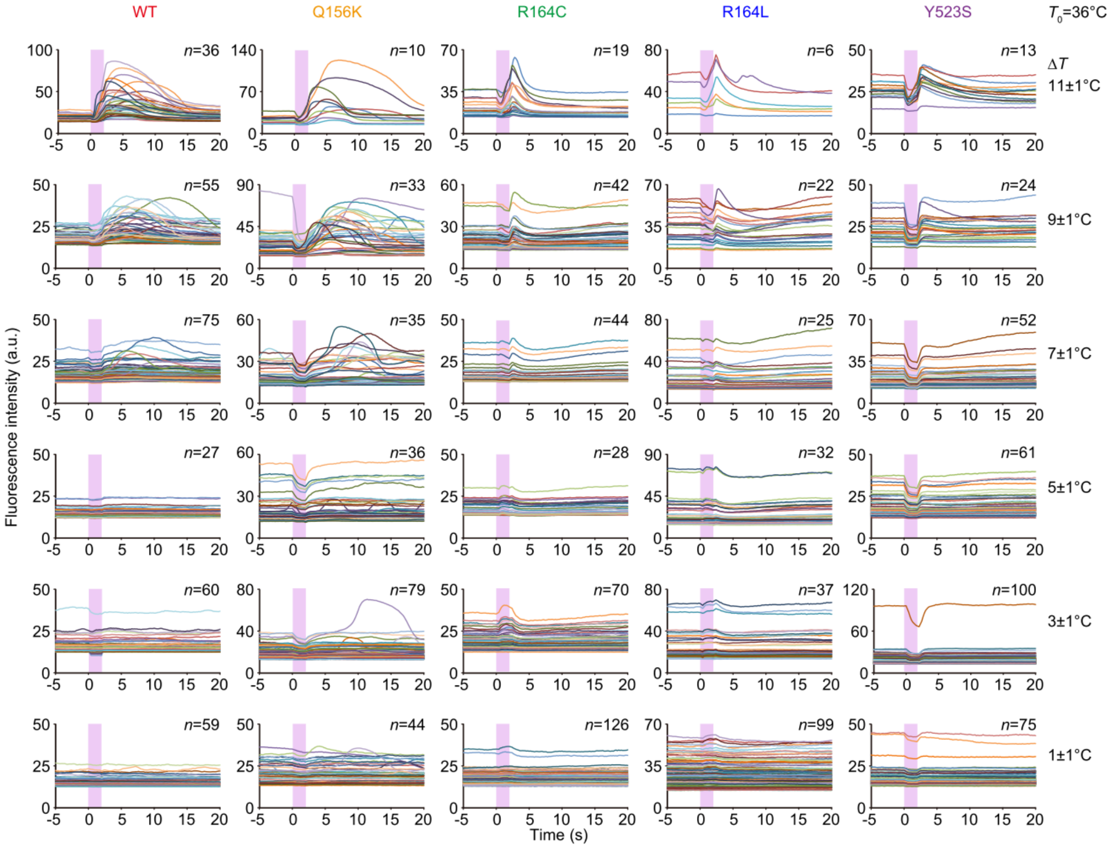

Figure S6. Intracellular Ca^{2+} response to a heat pulse of various amplitudes in individual cells at physiological temperature. Time course of changes in fluorescence intensity of fluo-4 in HEK293 cells expressing wild-type (WT) ryanodine receptor type 1 (RyR1) (left column) or RyR1 mutants at T_0=36°C. Changes in the background intensities caused by IR laser beam scattering were subtracted from the fluo-4 signals. Pink vertical bars indicate the period of heating.

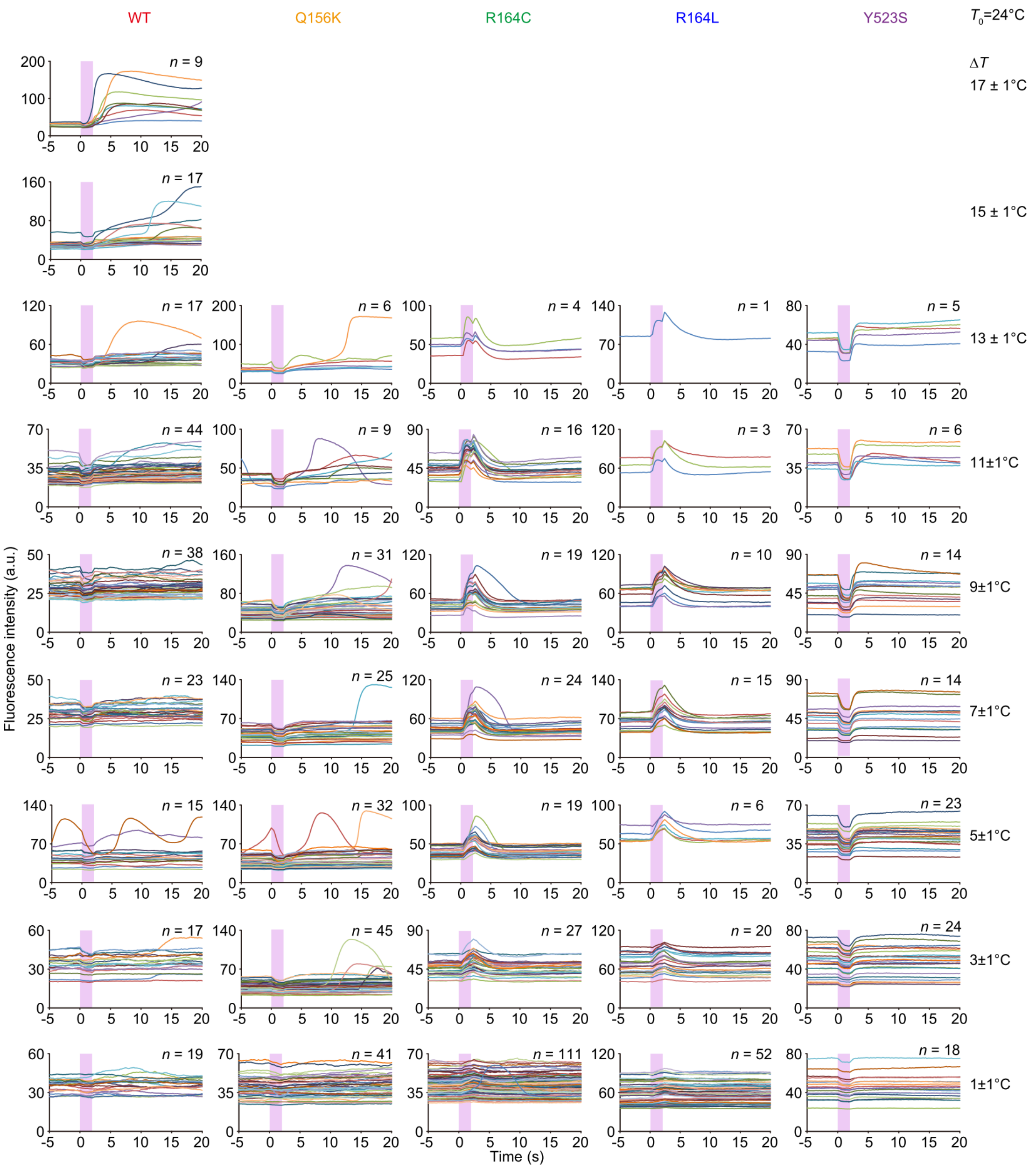

Figure S7. Intracellular Ca^{2+} response to a heat pulse of various amplitudes in individual cells at room temperature. Time course of changes in the fluorescence intensity of fluo-4 in HEK293 cells expressing wild-type (WT) ryanodine receptor type 1 (RyR1) (left column) or RyR1 mutants at T_0=24°C. Changes in the background intensities caused by IR laser beam scattering were subtracted from the fluo-4 signals. Pink vertical bars indicate the period of heating.

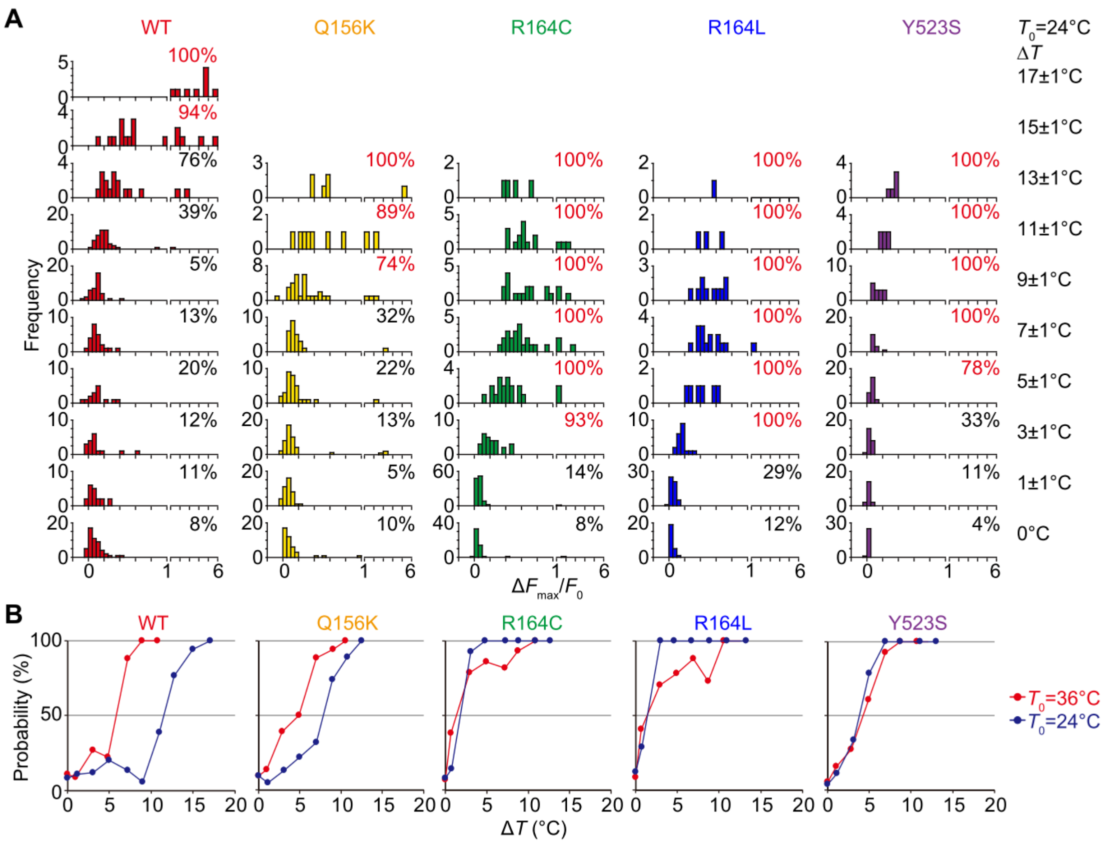

Figure S8. Intracellular Ca^{2+} response to a heat pulse of various amplitudes. (**A**) Histograms showing $[Ca^{2+}]_i$ increases ($\Delta F_{max}/F_0$ of fluo-4) in response to heating of various ΔT. Number in top right of each panel indicates the response probability of cells showing significant $[Ca^{2+}]_i$ increases ($\Delta F_{max}/F_0 > \Delta F_{th}$). Data in ***SI Appendix*, Fig. S7** were analyzed and plotted. T_0=24°C. (**B**) Relationship between ΔT and the response probability for various types of cells at T_0=24°C (blue line) and 36°C (red line).

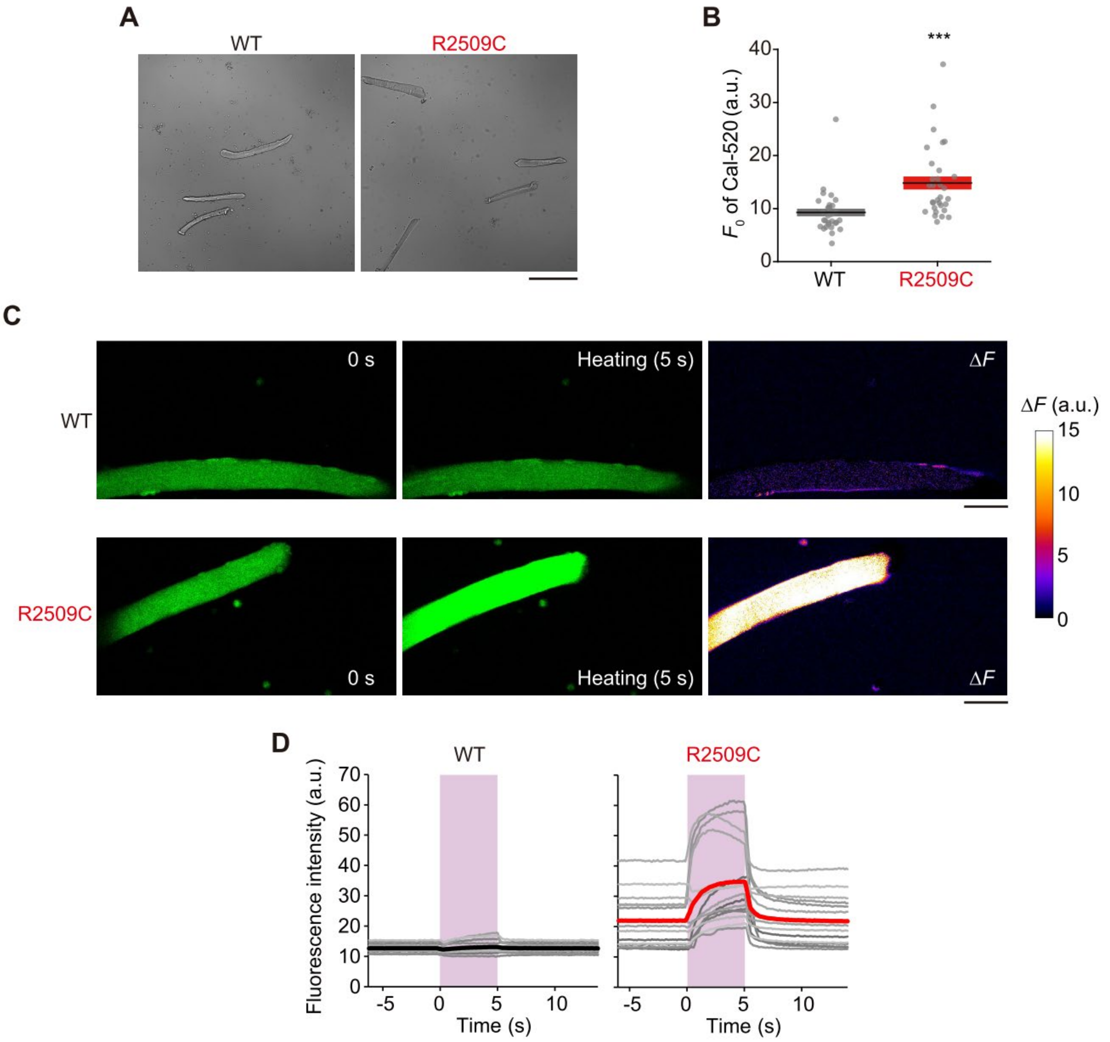

Figure S9. Heat-induced Ca^{2+} bursts in skeletal muscles expressing ryanodine receptor type 1 mutant R2509C. (**A**) Bright-field images of flexor digitorum brevis muscles isolated from wild-type (WT) (left) and R2509C (right) mice. Scale bar, 300 µm. (**B**) Fluorescence intensity of Cal-520 without heating, i.e., F_0. Number of cells, 31 for both WT and R2509C. Horizontal bars and boxes indicate means ± SEM. Statistical significance was examined using the Mann–Whitney *U* test (***p<0.001). p=6.1 × 10^{-6}. (**C**) Fluorescence images of Cal-520-loaded muscles from WT (top) or R2509C mice (bottom) before (left; t=0 s) and during heating (center; t=5 s). Figures on right indicate the differences in fluorescence intensity between t=0 and 5 s (ΔF) for WT (top) and R2509C

(bottom) muscles. ΔT=9°C; T_0=23°C. Scale bars, 50 µm. (**D**) Time course of changes in the fluorescence intensity of Cal-520-loaded muscles. Each gray line represents data for an individual muscle cell. Thick colored lines indicate average intensities. Pink vertical bars indicate the period of heating. ΔT=9±1°C; T_0=23°C.

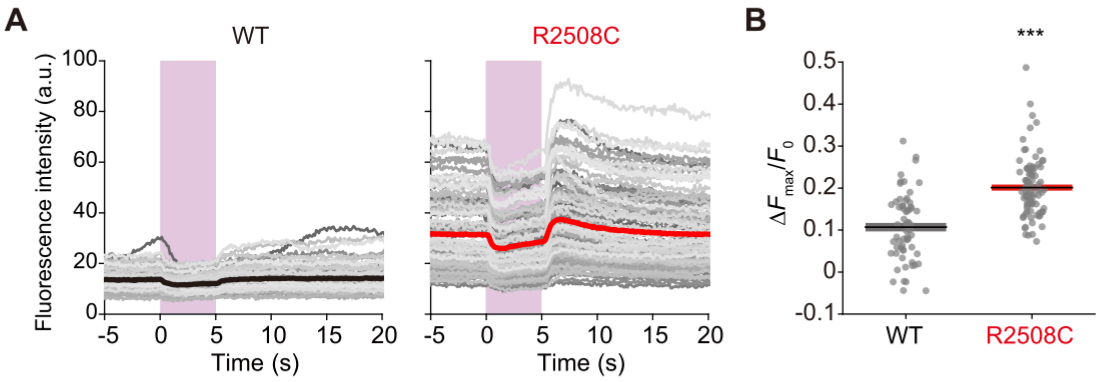

Figure S10. Heat-induced Ca^{2+} bursts in HEK293 cells expressing ryanodine receptor type 1 mutant R2508C. (A) Time course of the fluorescence intensity of Cal-520-loaded HEK293 cells expressing WT (left) or R2508C (right). Gray lines represent individual cells. Thick black and red lines indicate average intensities. Pink vertical bars indicate the period of heating. ΔT=9±1°C; T_0=25°C. (B) Maximal changes in relative fluorescence intensity of Cal-520 $\Delta F_{max}/F_0$ during the 20 s after the onset of heating. Horizontal bars and boxes indicate means ± SEM. Statistical significance was determined using the Mann–Whitney *U* test (****p*<0.001). WT, *n*=63; R2508C, *n*=92; and *p*=1.7 × 10^{-11}.

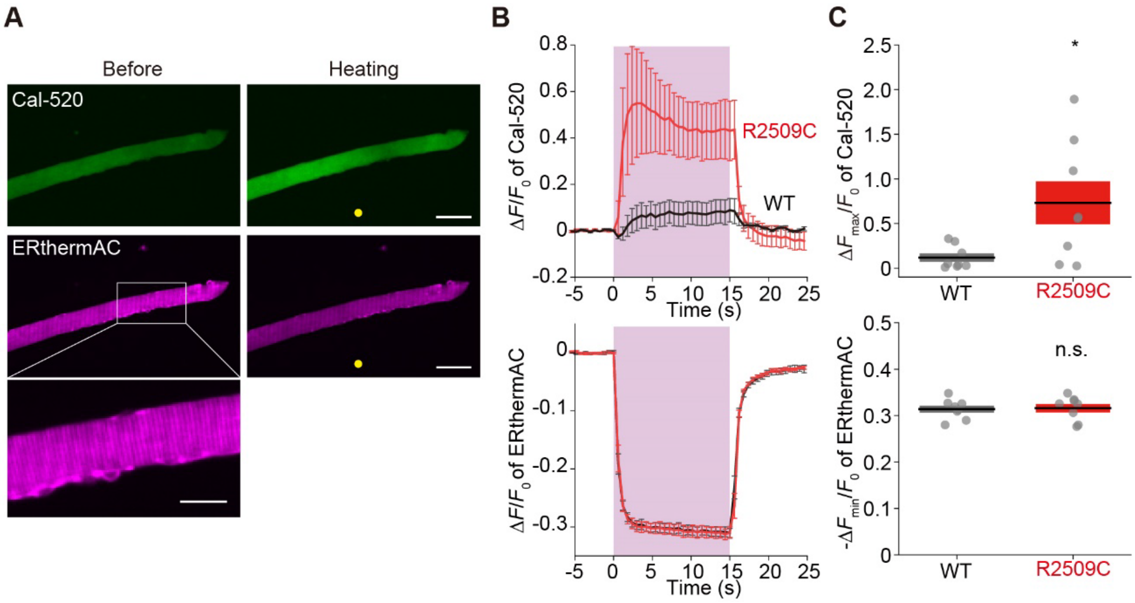

Figure S11. Temperature measurement of the SR in skeletal muscles during HICR. (A) Fluorescence images of flexor digitorum brevis muscles isolated from a R2509C mouse stained with Cal-520 (top) and the ER/SR-targeted luminescent molecular thermometer ERthermAC (middle) before (left; averaged image of 10 frames between -5.4 and 0 s) and during heating (right; averaged image of 10 frames between 9.6 and 15 s). Fluorescence intensity of ERthermAC decreases as the temperature rises. Yellow circles during heating indicate the position of the heat source. ΔT=9.5±0.5°C, T_0=25°C. Scale bars, 50 µm. Figure on the bottom left is the enlarged view of ERthermAC before heating. Scale bar, 20 µm. (B) Time course of a change in the fluorescence intensity of Cal-520 (top) and ERthermAC (bottom) in muscles isolated from WT or R2509C mice. Means ± SEM (n=8 for both WT and R2509C). Pink vertical bars indicate the period of heating. (C) Top, maximal increase in relative fluorescence intensity of Cal-520 ($\Delta F_{max}/F_0$) in muscles isolated from WT vs. R2509C mice during 25 s after the onset of heating. Bottom, maximal decrease in relative fluorescence intensity of ERthermAC ($\Delta F_{min}/F_0$) in muscles isolated from WT vs. R2509C mice during 25 s after the onset of heating. Horizontal bars

and boxes indicate means ± SEM. Statistical significance was determined by using the Mann–Whitney *U* test (*$p<0.05$; n.s., not significant). $n=8$ for both WT and R2509C. $p=0.038$ and 0.88 for Cal-520 and ERthermAC, respectively.

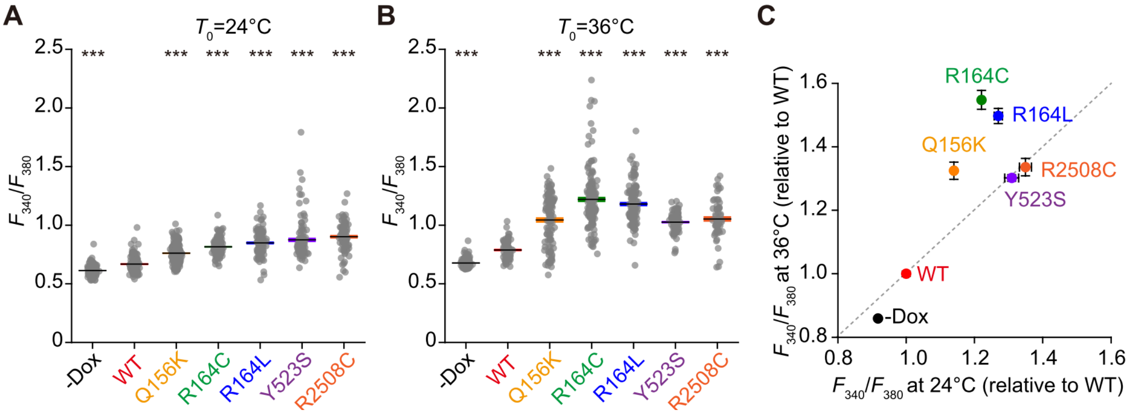

Figure S12. Temperature dependence of resting Ca^{2+} concentration in the cytosol in HEK293 cells. (**A, B**) The ratio of fluorescence intensity of fura-2 excited at 340 nm (F_{340}) to that at 380 nm (F_{380}) in HEK293 cells without induction of RyR1 expression [−doxycycline (−Dox)] as a control, or with induced expression of WT RyR1, or the mutants (Q156K, R164C, R164L, Y523S or R2508C) at 24°C (A) or 36°C (B). Horizontal bars and boxes indicate means ± SEM. Statistical significance was determined by comparison with WT using the Steel test (***p<0.001). At 24°C, -Dox, n=220; WT, n=126; Q156K, n=251; R164C, n=183; R164L, n=116; Y523S, n=111; R2508C, n=87. At 36°C, -Dox, n=118; WT, n=91; Q156K, n=112; R164C, n=152; R164L, n=115; Y523S, n=99; R2508C, n=61. (**C**) Comparison of resting $[Ca^{2+}]_i$ between 24°C and 36°C. Ratio values at each temperature were normalized to that of WT. A dashed line indicates that the relative ratio value at 36°C is equal to that at 24°C. Means ± SEM.

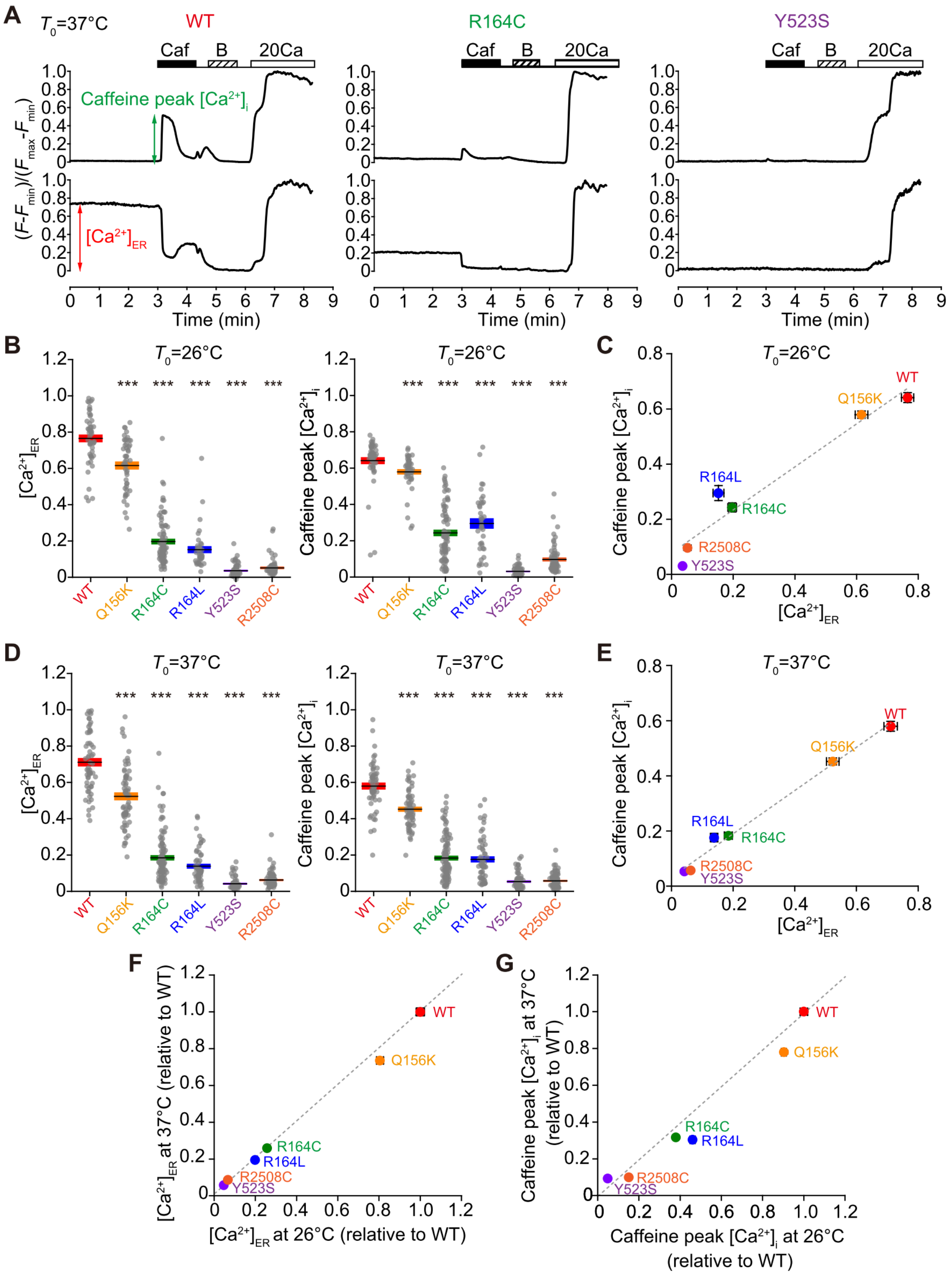

Figure S13. Temperature dependence of the Ca^{2+} concentration in the ER in HEK293 cells. (**A**) Representative traces of G-GECO1.1 (upper) and R-CEPIA1*er* (lower) signals in WT (left), R164C (middle), and Y523S (right) cells at 37˚C. G-GECO1.1 was expressed in the cytosol, and R-CEPIA1*er* targeted to the ER. Ca^{2+} signals in individual cells were

obtained in normal Krebs solution and then in 10 mM caffeine solution (thick line). At the end of each measurement, the cells were perfused with 0Ca-Krebs solution (thin line), BAPTA-ionomycin solution (hatched bar), 0Ca-Krebs solution (thin line), and then 20Ca-ionomycin solution (open bar). F_{min} and F_{max} were the values at BAPTA-ionomycin and 20Ca-ionomycin solutions, respectively. Peak $[Ca^{2+}]_i$ transients indicated by a green double arrow and resting $[Ca^{2+}]_{ER}$ by a red double arrow were analyzed. (**B**) Resting $[Ca^{2+}]_{ER}$ in normal Krebs solution (left) and peak $[Ca^{2+}]_i$ transients in response to 10 mM caffeine (right) in WT and mutant cells at 26˚C. Horizontal bars and boxes indicate means ± SEM. Statistical significance was determined by comparison with WT using the Steel test (***p<0.001). Resting $[Ca^{2+}]_{ER}$: WT, n=50; Q156K, n=49; R164C, n=82; R164L, n=39; Y523S, n=70; R2508C, n=65. Peak $[Ca^{2+}]_i$ transients: WT, n=49; Q156K, n=49; R164C, n=82; R164L, n=38; Y523S, n=69; R2508C, n=65. (**C**) Comparison of resting $[Ca^{2+}]_{ER}$ and peak $[Ca^{2+}]_i$ transients in response to 10 mM caffeine at 26˚C. R = 0.971 (p=1.2 × 10^{-3}). (**D**) Resting $[Ca^{2+}]_{ER}$ in normal Krebs solution (left) and peak $[Ca^{2+}]_i$ transients in response to 10 mM caffeine (right) in WT and mutant cells at 37˚C. Horizontal bars and boxes indicate means ± SEM. Statistical significance was determined by comparison with WT using the Steel test (***p<0.001). Resting $[Ca^{2+}]_{ER}$: WT, n=58; Q156K, n=65; R164C, n=102; R164L, n=58; Y523S, n=68; R2508C, n=84. Peak $[Ca^{2+}]_i$ transients: WT, n=55; Q156K, n=65; R164C, n=102; R164L, n=58; Y523S, n=68; R2508C, n=84. (**E**) Comparison of resting $[Ca^{2+}]_{ER}$ and peak $[Ca^{2+}]_i$ transients in response to 10 mM caffeine at 37˚C. Correlation coefficient (R) was 0.995 (p=3.1 × 10^{-5}). (**F, G**) Comparison of resting $[Ca^{2+}]_{ER}$ (**F**) or peak $[Ca^{2+}]_i$ transients in response to 10 mM caffeine (**G**) between 26°C and 37°C. At both temperatures, the ratio values were normalized to that of WT. A dashed line indicates that the relative ratio value at 37°C is equal to that at 26°C. Means ± SEM.

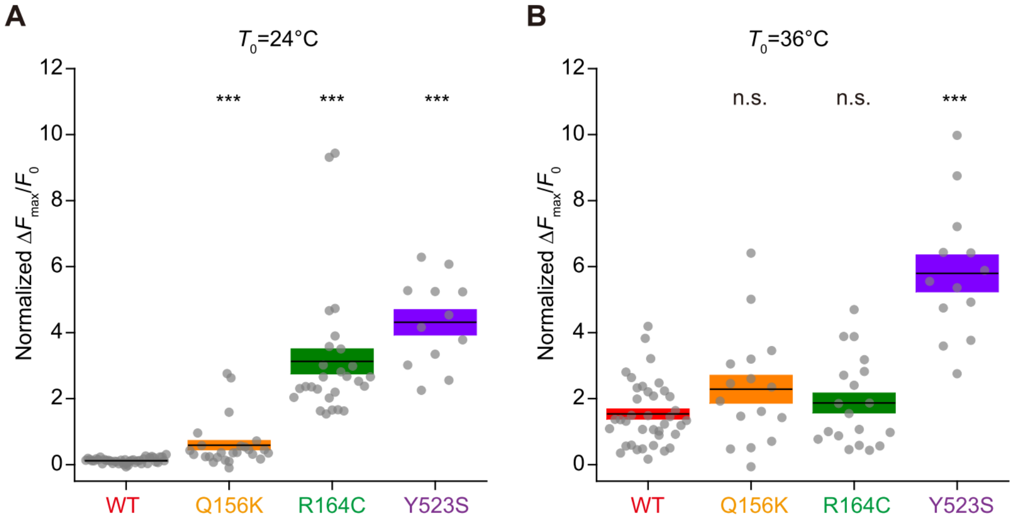

Figure S14. Normalized amplitudes of Ca^{2+} bursts in HEK293 cells expressing ryanodine receptor type 1 mutants. (**A**) Maximal changes in the relative fluorescence intensity of fluo-4 $\Delta F_{max}/F_0$ in **Fig. 2D** following normalization by the relative resting $[Ca^{2+}]_{ER}$ of each mutant to that of WT (***SI Appendix*, Fig. S13B**). $\Delta F_{max}/F_0$ was divided by the relative resting $[Ca^{2+}]_{ER}$ compared to WT; i.e., 0.805, 0.257, and 0.047 for Q156K, R164C, and Y523S, respectively. Horizontal bars and boxes indicate means ± SEM. Statistical significance was determined by comparison with WT cells (n=43) using the Steel test (***p<0.001). Q156K, n=25 and $p=1.4 \times 10^{-6}$; R164C, n=27 and $p=6.1 \times 10^{-12}$; Y523S, n=12 and $p=4.4 \times 10^{-7}$. Laser power, 25.6 mW; ΔT=10±2°C; T_0=24°C. (**B**) Maximal changes in the relative fluorescence intensity of fluo-4 $\Delta F_{max}/F_0$ in **Fig. 2F** following normalization by the relative resting $[Ca^{2+}]_{ER}$ of each mutant to that of WT (***SI Appendix*, Fig. S13D**). $\Delta F_{max}/F_0$ was divided by the relative resting $[Ca^{2+}]_{ER}$ compared to WT; i.e., 0.736, 0.260, and 0.059 for Q156K, R164C, and Y523S, respectively. Horizontal bars and boxes indicate means ± SEM. Statistical significance was determined by comparison with WT cells (n=38) using the Steel test (***p<0.001; n.s., not significant). Q156K, n=16 and p=0.28; R164C, n=19 and p=0.89; Y523S, n=13 and $p=7.1 \times 10^{-7}$. Laser power, 25.6 mW; ΔT=10±1°C; T_0=36°C.

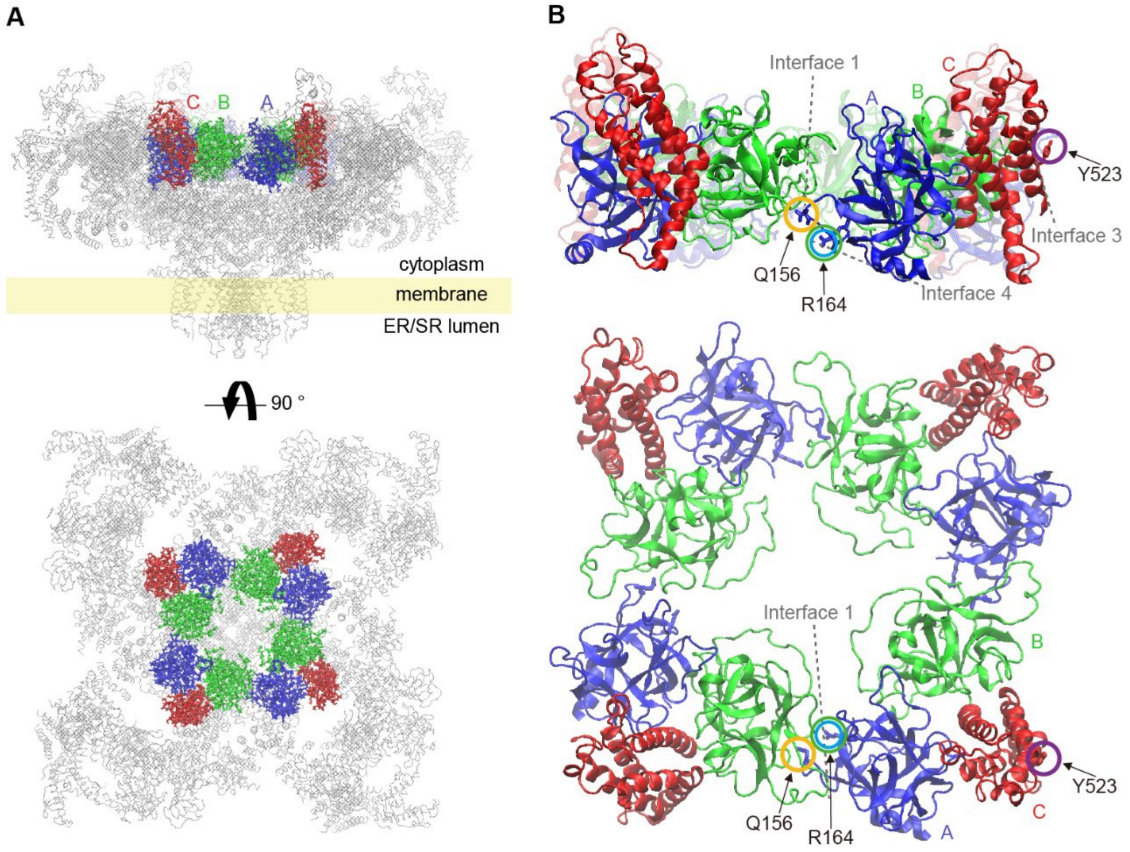

Figure S15. Structure of ryanodine receptor type 1. (**A**) Overall view of ryanodine receptor type 1 (PDB ID: 5GKZ) (1) structure. Side view (top), and top view from the cytoplasm (bottom). The N-terminal domain (1–532, NTD) tetramer is colored. The subdomains (A, B, and C) are shown in blue, green, and red, respectively, as reported by Tung et al. (PDB ID: 2XOA) (2). (**B**) Enlarged view of the NTD tetramer. Side view (top), and top view from the cytoplasm (bottom). Mutation sites (R164, Q156, and Y523) and interacting interfaces (2) are labeled. The structure of amino-acid residues of mutation sites is displayed as a licorice model. Interfaces 2, 5, and 6 are not shown because the mutations used in the present study (R164, Q156, and Y523) are not located therein.

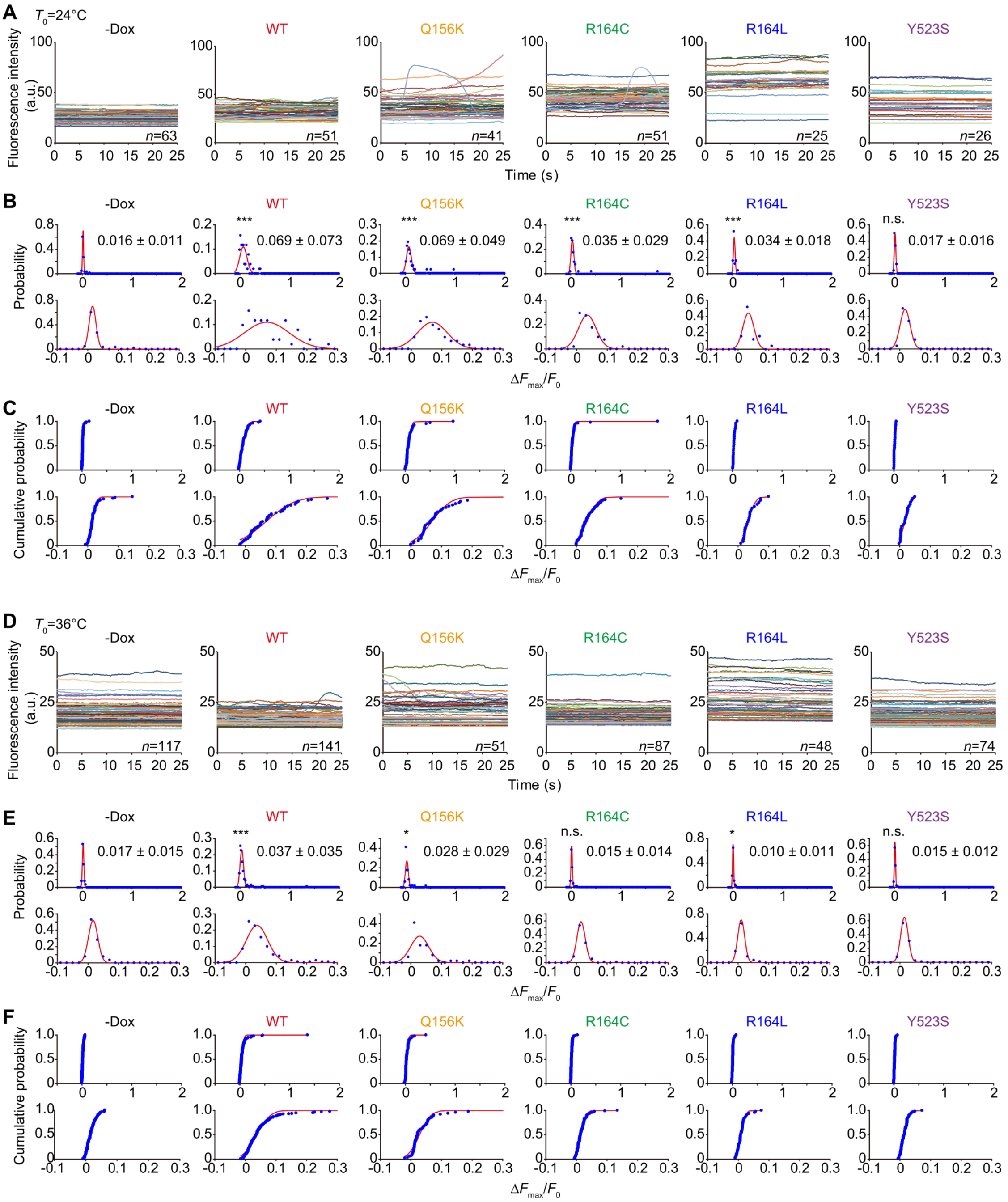

Figure S16. Endogenous fluctuations of $[Ca^{2+}]_i$ in HEK293 cells expressing ryanodine receptor type 1 mutants. (**A**) Time course of changes in relative fluorescence intensity $\Delta F/F_0$ of fluo-4 in HEK293 cells with or without induced expression of ryanodine receptor type 1. T_0=24°C. (**B** and **C**) Histograms (**B**) and the cumulative histograms (**C**) for maximum changes in relative fluorescence intensity ($\Delta F_{max}/F_0$) of fluo-4. Data in (**A**) were

analyzed and plotted. Data (blue plots) were fitted by a single Gaussian function (**B**) or by a cumulative distribution function of the Gaussian distribution (**C**) (red lines). See **Materials and Methods** for details of the fitting procedures. Numbers in (**B**) indicate means ± SD, which were used to determine ΔF_{th} (see **Materials and Methods**). (**D**) Same as in (**A**) but T_0=36°C. (**E** and **F**) Same as in (**B**) and (**C**), respectively, but using the data in (**D**). In (**B**) and (**E**), statistical significance was examined with cells without induction of RyR1 expression [−doxycycline (−Dox)] using the Steel test (**p*<0.05; ****p*<0.001; n.s., not significant). In (**B**), $p=3.0 \times 10^{-6}$ [wild-type (WT)], 6.4×10^{-11} (Q156K), 2.3×10^{-4} (R164C), 3.0×10^{-5} (R164L), and 1.00 (Y523S). In (**E**), $p=2.8 \times 10^{-7}$ (WT), 0.028 (Q156K), 0.81 (R164C), 0.032 (R164L), and 0.87 (Y523S).

Supplemental Results and Discussion

Heat-induced Ca^{2+} release changes resting $[Ca^{2+}]$ in the cytosol and ER

We compared resting $[Ca^{2+}]_i$ using the ratiometric fluorescent Ca^{2+} indicator fura-2 at 24°C vs. 36°C in various cells (***SI Appendix*, Fig. S12A and B**). All the mutant cells demonstrated significantly higher $[Ca^{2+}]_i$ compared with WT cells (~1.2 times or higher) in both conditions (***SI Appendix*, Fig. S12A and B**). However, it is not reasonable to directly compare the ratio values measured at different temperatures, owing to some effects on the Ca^{2+} measurement (e.g., temperature change and pH). Therefore, we calculated the ratio values normalized to those obtained in WT cells at either temperature, and compared the normalized ratio values between various cells to evaluate the effects of a temperature rise on $[Ca^{2+}]_i$ (***SI Appendix*, Fig. S12C**). We found an increase in $[Ca^{2+}]_i$ upon a temperature change from 24°C to 36°C in Q156K (from 1.1 to 1.3 times), R164C (from 1.2 to 1.5), and R164L (from 1.3 to 1.5) cells compared with WT cells (***SI Appendix*, Fig. S12C**). The higher heat sensitivity of these mutant cells is consistent with our findings that heat-induced Ca^{2+} release is elicited more easily in the mutants than WT cells. The $[Ca^{2+}]_i$ level in Y523S or R2508C cells was approximately 1.3 times higher than in WT cells at both 24°C and 36°C. It is to be noted that the enhanced heat sensitivity is not apparent, because these cells reportedly demonstrate a relatively high $[Ca^{2+}]_i$ and low $[Ca^{2+}]$ in the ER ($[Ca^{2+}]_{ER}$) in previous studies (3, 4). Should $[Ca^{2+}]_{ER}$ be very low at 24°C, $[Ca^{2+}]_i$ would remain similar at 36°C, even though Ca^{2+} release is accelerated from the ER. This possibility can be further tested by the caffeine assay and the direct measurement of $[Ca^{2+}]_{ER}$ using ER-targeted fluorescent Ca^{2+} probes. Accordingly, we compared resting $[Ca^{2+}]_{ER}$ and peak $[Ca^{2+}]_i$ transients in response to 10 mM caffeine at 26˚C and 37˚C (***SI Appendix*, Fig. S13A-E**). The $[Ca^{2+}]_i$ and $[Ca^{2+}]_{ER}$ signals were simultaneously determined using the genetically encoded Ca^{2+} indicators, G-GECO1.1 and R-CEPIA1*er*, respectively (***SI Appendix*, Fig. S13A**). All the mutant cells demonstrated significantly lower $[Ca^{2+}]_{ER}$ levels compared with WT cells at both temperatures (***SI Appendix*, Fig. S13B, D**). Peak $[Ca^{2+}]_i$ transients induced by the caffeine application, which indicates $[Ca^{2+}]_{ER}$, were likewise lower in mutant cells than WT (***SI***

Appendix*, Fig. S13B, D**). Resting $[Ca^{2+}]_{ER}$ and peak $[Ca^{2+}]_i$ transients were correlated well at both temperatures (SI Appendix*, Fig. S13C, E**). Normalized values of $[Ca^{2+}]_{ER}$ and the peak $[Ca^{2+}]_i$ to WT cells at 26˚C and 37˚C were again compared (***SI Appendix*, Fig. S13F and G**). Consistent with the $[Ca^{2+}]_i$ level in Q156K cells that was elevated from 24°C to 36°C (***SI Appendix*, Fig. S13C**), resting $[Ca^{2+}]_{ER}$ in these cells compared to those in WT decreased from 0.8 to 0.7 times in association with an increase in temperature from 26˚C to 37˚C (***SI Appendix*, Fig. S13F**). Likewise, peak $[Ca^{2+}]_i$ transients decreased from 0.9 to 0.8 times in association with an increase in temperature from 26˚C to 37˚C (***SI Appendix*, Fig. S13G**). The values in R164C and R164L cells were low at both temperatures (***SI Appendix*, Fig. S13A, B, D**). Although the effect of temperature was not clear on resting $[Ca^{2+}]_{ER}$ (***SI Appendix*, Fig. S13F**), in these cells, peak $[Ca^{2+}]_i$ transients tended to be lowered when temperature was increased from 26˚C to 37˚C (***SI Appendix*, Fig. S13G**). Both resting $[Ca^{2+}]_{ER}$ and peak $[Ca^{2+}]_i$ transients were extremely low in Y523S and R2508C cells, as expected (***SI Appendix*, Fig. S13A, B, D**); these findings made it difficult to quantify the effects of temperature on the above-mentioned values.

Supplemental Materials and Methods

Ca^{2+} imaging of skeletal muscles

Isolated single flexor digitorum brevis (FDB) cells from WT and R2509C mice (3–8 months old) were seeded on an iMatrix-511 (T311; Takara Bio, Shiga, Japan)-coated cover slip of the 35 mm glass base dish (3960-035; AGC Techno Glass, Shizuoka, Japan) for Ca^{2+} imaging. The single FDB cells were incubated with 4 μM Cal-520-AM dye (21130; AAT BioQuest, San Francisco, CA) in HEPES-Krebs solution containing 1 mg/mL bovine serum albumin (BSA) for 40–50 min at 37°C. The cells were then washed three times with the HEPES-Krebs solution to remove excess dye. After 30 min of de-esterification, fluorescence images were obtained with a 20× objective lens (UPlanSApo, NA=0.75; Olympus, Tokyo, Japan) of an inverted microscope (IX73; Olympus) equipped with a Nipkow confocal scanner unit (CSU-X1; Yokogawa Electric, Tokyo, Japan), a dichroic mirror (Di01-T405/488/561; Semrock, Rochester, NY), and an EM-CCD camera (iXon Ultra; Andor Technology, Belfast, UK). Cal-520 was excited by a 488 nm laser light (Vortran Laser Technology, Sacramento, CA), and the fluorescence through an emission filter (FF01-520/35; Semrock) was captured. Local temperature was increased by an infra-red laser (KPS-STD-BT-RFL-1455-05-CO; Keopsys, Lannion, France) with IR-LEGO mini (SIGMAKOKI, Tokyo, Japan). IR laser beam scattering was negligible in this confocal imaging system (**Movie S6**). The fluorescence intensities of Cal-520 in the area of 314 μm^2 were measured. To minimize artifacts caused by the movement and drifting focus due to contracture during heating, the area at the center of the cell was selected. The change in the fluorescence intensity (ΔF) of Cal-520 was calculated from $F - F_{before}$, where F was the fluorescence intensity at an arbitrary time, and F_{before} was the averaged intensity for 1.2 s just before the observation was initiated (i.e., 10 s before beginning the heating). The basal fluorescence intensity of Cal-520 (F_0) was calculated from $F_{before} - I_{back}$, where I_{back} was the background intensity when the excitation light was off. The peak intensity of Cal-520 ($\Delta F_{max}/F_0$) was calculated from the maximum $\Delta F/F_0$ during the ~30 s after heating initiation.

The change in local temperature was calculated by thermal quenching of 10 μg/mL Alexa Fluor 555 conjugated to 10 kDa dextran (D34679; Thermo Fisher Scientific, Waltham, MA) in HEPES-Krebs solution based on a previously described procedure (5). The dye was excited by a 561 nm laser light (Cobolt, Solna, Sweden), and the fluorescence through an emission filter (FF01-617/73; Semrock) was captured. These microscopic experiments were performed at 23±0.5°C and 36.5±0.5°C.

Temperature measurement of skeletal muscles

The single FDB cells from WT and R2509C mice (14 weeks old) were incubated with 4 μM Cal-520-AM and 100 nM ERthermAC in HEPES-Krebs solution containing 1 mg/mL BSA for 30–40 min at room temperature. ERthermAC is a photostable luminescent molecular thermometer that selectively targets the ER/SR (6, 7). After washing three times with the HEPES-Krebs solution and de-esterification for 30 min, FDB cells in HEPES-Krebs solution containing 100 μM BTS (N-Benzyl-p-toluenesulfonamide) (B3082; Tokyo Chemical Industry, Tokyo, Japan) were observed under the same confocal microscope as that used for Ca^{2+} imaging in FDB cells. ERthermAC was excited by the 561 nm laser light, and the fluorescence through an emission filter (FF01-617/73) was captured. These microscopic experiments were performed at 25°C. Excitation light and emission filters for Cal-520 and ERthermAC were exchanged alternately. Exposure time was 100 ms for both images, with a lag time of 200 ms between images.

Ca^{2+} imaging of HEK293 cells expressing R2508C

HEK293 cells expressing wild-type RyR1 or R2508C were incubated with 4 μM Cal-520-AM in HEPES-Krebs solution containing 1 mg/mL BSA for 30–40 min at 37°C. After washing three times with the HEPES-Krebs solution and de-esterification for 30 min, fluorescence images were obtained with the same confocal microscope as used for Ca^{2+} imaging in FDB cells. Changes in the fluorescence intensities of Cal-520 were analyzed by the same method as that for fluo-4.

Western blotting

HEK293 cells expressing WT and mutant RyR1 were plated on tissue culture dishes, and protein expression was induced by doxycycline (2 µg/mL) for 24 h. The cells were harvested and rinsed twice with phosphate-buffered saline. The total protein was extracted, separated by sodium dodecyl sulfate polyacrylamide gel electrophoresis, and transferred onto a polyvinylidene fluoride membrane. Western blotting was performed using antibodies for RyR1 (34C; Developmental Studies Hybridoma Bank, University of Iowa, Iowa City, IA), SERCA2 (sc-376235; Santa Cruz Biotechnology, Dallas, TX) and calnexin (C4731; Sigma-Aldrich, St. Louis, MO).

Resting cytosolic Ca^{2+} measurements

HEK293 cells were incubated with 4 µM fura-2-AM (Thermo Fisher Scientific) dye in HBS solution, containing 1 mg/mL BSA, for 30 min at room temperature (23–26°C). The cells were then washed three times with HBS solution to remove excess dye. After 30 min of de-esterification, fluorescence images were obtained with a 20× objective lens (NA 0.75, UPlanSApo, Olympus) under an inverted microscope (DMI6000B, Leica, Wetzlar, Germany) equipped with a complementary metal-oxide-semiconductor camera (ORCA-Flash4.0, Hamamatsu Photonics, Hamamatsu, Japan), operating at 0.5 frame/s, using a MetaFluor v7 imaging system (Molecular Devices, Sunnyvale, CA) at 24±0.2°C and then 36±0.5°C. The fluorescence wavelength was switched by using a filter wheel (LAMBDA 10-3, Sutter Instrument, Novato, CA). For fura-2, it was excited at alternating wavelengths of 340 (340±13) nm and 380 (387±6) nm, and the fluorescence was measured at 520±14 nm for both wavelengths. Regions of interest corresponding to individual cells were selected, and the mean fluorescence intensity (F) of each region of interest minus the background intensity was calculated for each frame. We used the F_{340}/F_{380} ratio (the value of F at 340 nm excitation divided by the value at 380 nm excitation) to compare $[Ca^{2+}]_i$, as described previously (4, 8).

Simultaneous measurements of cytosolic and ER [Ca^{2+}] in HEK293 cells

[Ca^{2+}]$_i$ and [Ca^{2+}]$_{ER}$ were simultaneously measured using the genetically encoded Ca^{2+} indicators, i.e., G-GECO1.1 [expressed in the cytosol (9)] and R-CEPIA1*er* [targeted to the ER (3, 10)], respectively. Cells were transfected with G-GECO1.1 and R-CEPIA1*er* cDNA for 24 h before measurements. Doxycycline was added to the medium at the same time of transfection. G-GECO1.1 and R-CEPIA1*er* were excited by 488 nm and 568 nm light, respectively, and fluorescence images at 525 and 620 nm were simultaneously captured using the W-view system (Hamamatsu Photonics) and an EM-CCD camera (Model 8509, Hamamatsu Photonics). The [Ca^{2+}]$_i$ and [Ca^{2+}]$_{ER}$ signals were measured in normal Krebs solution (140 mM NaCl, 5 mM KCl, 2 mM $CaCl_2$, 1 mM $MgCl_2$, 11 mM glucose, 10 mM HEPES, pH 7.4) for 3 min and then in caffeine-Krebs solution (Krebs solution containing 10 mM caffeine) for 1.2 min. The cells were then bathed in the following solutions: i.e., 0Ca-Krebs solution (140 mM NaCl, 5 mM KCl, 1 mM $MgCl_2$, 11 mM glucose, 10 mM HEPES, pH 7.4), BAPTA-0Ca-ionomycin solution (140 mM NaCl, 5 mM KCl, 5 mM 1,2-bis(o-aminophenoxy)ethane-N,N,N',N'-tetraacetic acid (BAPTA), 1 mM $MgCl_2$, 11 mM glucose, 0.02 mM ionomycin, 0.02 mM cyclopiazonic acid, 10 mM HEPES, pH 7.4), 0Ca-Krebs solution, and finally 20Ca-ionomycin solution (140 mM NaCl, 5 mM KCl, 20 mM $CaCl_2$, 1 mM $MgCl_2$, 11 mM glucose, 0.02 mM ionomycin, 10mM HEPES, pH 7.4). F_{min} and F_{max} were determined in the BAPTA-0Ca-ionomycin solution and 20Ca-ionomycin solution, respectively, and the fluorescence signals (F - F_{min}) in normal- and caffeine-Krebs solutions were normalized by the maximal fluorescence changes (F_{max} - F_{min}) of the Ca^{2+} indicators.

Supplemental References

1. X. C. Bai, Z. Yan, J. Wu, Z. Li, N. Yan, The Central domain of RyR1 is the transducer for long-range allosteric gating of channel opening. *Cell Res.* **26**, 995–1006 (2016).
2. C.-C. Tung, P. A. Lobo, L. Kimlicka, F. Van Petegem, The amino-terminal disease hotspot of ryanodine receptors forms a cytoplasmic vestibule. *Nature* **468**, 585–588 (2010).
3. T. Murayama, *et al.*, Divergent activity profiles of type 1 ryanodine receptor channels carrying malignant hyperthermia and central core disease mutations in the amino-terminal region. *PLoS One* **10**, e0130606 (2015).
4. T. Murayama, *et al.*, Genotype-phenotype correlations of malignant hyperthermia and central core disease mutations in the central region of the RYR1 channel. *Hum. Mutat.* **37**, 1231–1241 (2016).
5. K. Oyama, *et al.*, Directional bleb formation in spherical cells under temperature gradient. *Biophys. J.* **109**, 355–364 (2015).
6. R. Kriszt, *et al.*, Optical visualisation of thermogenesis in stimulated single-cell brown adipocytes. *Sci. Rep.* **7**, 1383 (2017).
7. Y. Hou, *et al.*, Ca^{2+}-associated triphasic pH changes in mitochondria during brown adipocyte activation. *Mol. Metab.* **6**, 797–808 (2017).
8. T. Yamazawa, *et al.*, Insights into channel modulation mechanism of RYR1 mutants using Ca^{2+} imaging and molecular dynamics. *J. Gen. Physiol.* **152**, e201812235 (2020).
9. Y. Zhao, *et al.*, An expanded palette of genetically encoded Ca^{2+} indicators. *Science* **333**, 1888–1891 (2011).
10. J. Suzuki, *et al.*, Imaging intraorganellar Ca^{2+} at subcellular resolution using CEPIA. *Nat. Commun.* **5**, 4153 (2014).

Legends for Supplemental Movies

Movie S1. Ca^{2+} response to a heat pulse in HEK293 cells expressing wild-type ryanodine receptor type 1. Fluorescence microscope images of fluo-4-loaded HEK293 cells expressing wild-type ryanodine receptor type 1. Scale bar, 20 µm; laser power, 25.6 mW; T_0=24°C.

Movie S2. Ca^{2+} response to a heat pulse in HEK293 cells expressing R164C. Fluorescence microscope images of fluo-4-loaded HEK293 cells expressing R164C. Heat-induced Ca^{2+} bursts are apparent. Scale bar, 20 µm; laser power, 25.6 mW; T_0=24°C.

Movie S3. Ca^{2+} response to a heat pulse in HEK293 cells expressing Q156K. Fluorescence microscope images of fluo-4-loaded HEK293 cells expressing Q156K. Heat-induced Ca^{2+} bursts are apparent. Scale bar, 20 µm; laser power, 25.6 mW; T_0=24°C.

Movie S4. Ca^{2+} response to a heat pulse in HEK293 cells expressing Y523S. Fluorescence microscope images of fluo-4-loaded HEK293 cells expressing Y523S. Heat-induced Ca^{2+} bursts are apparent. Scale bar, 20 µm; laser power, 25.6 mW; T_0=24°C.

Movie S5. Heat-induced endoplasmic reticulum Ca^{2+} dynamics in HEK293 cells expressing wild-type ryanodine receptor type 1. Fluorescence microscope images of G-CEPIA1*er* in HEK293 cells expressing wild-type ryanodine receptor type 1. Scale bar, 20 µm; laser power, 25.6 mW; T_0=36°C.

Movie S6. Ca^{2+} response to a heat pulse in a skeletal muscle expressing wild-type ryanodine receptor type 1. Fluorescence microscope images of a Cal-520-loaded skeletal muscle expressing wild-type ryanodine receptor type 1. Scale bar, 50 µm; ΔT=3.5°C; T_0=23°C.

Movie S7. Ca^{2+} response to a heat pulse in a skeletal muscle expressing R2509C. Fluorescence microscope images of a Cal-520-loaded skeletal muscle expressing R2509C. Heat-induced Ca^{2+} bursts are apparent. Scale bar, 50 µm; ΔT=3.5°C; T_0=23°C.

Movie S8. Ca^{2+} response to a heat pulse in a skeletal muscle expressing wild-type ryanodine receptor type 1. Fluorescence microscope images of a Cal-520-loaded skeletal muscle expressing wild-type ryanodine receptor type 1. Scale bar, 50 µm; ΔT=9°C; T_0=23°C.

Movie S9. Ca^{2+} response to a heat pulse in a skeletal muscle expressing R2509C. Fluorescence microscope images of a Cal-520-loaded skeletal muscle expressing R2509C. Heat-induced Ca^{2+} bursts are apparent. Scale bar, 50 µm; ΔT=9°C; T_0=23°C.
